# FUT8 Catalysis Involves GDP-Fucose–Induced Loop Activation Promoting a Reaction at the S_N_1‑S_N_2 Frontier

**DOI:** 10.1021/acscatal.5c07826

**Published:** 2025-12-19

**Authors:** Ignacio Sanz-Martínez, Tomás Tejero, Ramón Hurtado-Guerrero, Pedro Merino

**Affiliations:** † Institute of Biocomputation and Physics of Complex Systems (BIFI), 16765 University of Zaragoza, Campus Rio Ebro, Zaragoza E-50018, Spain; ‡ Department of Organic Chemistry. Faculty of Sciences, University of Zaragoza, Campus San Francisco, Zaragoza E-50009, Spain; § Institute of Chemical Synthesis and Homogeneous Catalysis (ISQCH), University of Zaragoza-CSIC, Campus San Francisco, Zaragoza E-50009, Spain; ∥ Copenhagen Center for Glycomics, Department of Cellular and Molecular Medicine, University of Copenhagen, Blegdamsvej 3B, DK-2200 Copenhagen, Denmark; ⊥ Aragonese Foundation for Research & Development (ARAID), Government of Aragon, Avda. Ranillas, 1D. Zaragoza E-50018, Spain

**Keywords:** glycosyltransferases, loop activation, S_N_1-S_N_2 mechanism, metadynamics, transient glycosyl cations, FUT8, ELF analysis

## Abstract

α1,6-Fucosyltransferase 8 (FUT8) catalyzes the core α1,6-fucosylation of *N*-glycans, a modification essential for the biological function of many mammalian glycoproteins. Despite its importance, the structural and mechanistic aspects of FUT8 catalysis remain incompletely understood. Here, we combine molecular dynamics, QM/MM, and metadynamics simulations to delineate the full catalytic cycle of FUT8. We reveal that GDP-fucose binding induces a concerted conformational rearrangement of two flexible loops, which cooperatively stabilize a closed, catalytically competent active site. Formation of the Michaelis complex primes the enzyme for fucose transfer via a slightly late and highly asynchronous S_N_2 inverting mechanism. In fact, the reaction proceeds through a late transition state in a single kinetic step (energy barrier ∼18 kcal/mol, consistent with experimental *k*_cat_ values) but in three different stages, i.e.: (i) cleavage of the glycosidic bond between fucose and GDP, (ii) formation of the glycosidic bond and (iii) H-transfer from the acceptor to the catalytic Glu373, underscoring the asynchronous nature of this process. Moreover, topological calculations of the electron localization function (ELF) along the reaction coordinate reveal the transient formation of an intimate ion pair, with a lifetime of 350–800 fs, as a transient intermediate; notably, despite the cationic character of the transition state, no stable intermediate is formed. These findings highlight how structural rearrangements enable a chemically distinct catalytic process and provide a structural framework for rational inhibitor design.

## Graphical abstract

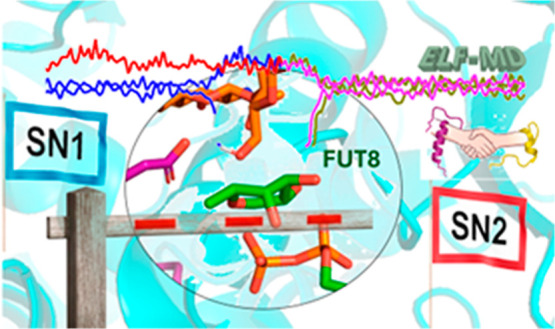

## Introduction

Fucosyltransferases (FUTs) play a fundamental role in cellular biology, catalyzing the transfer of fucosyl residues from GDP-β-l-fucose (GDP-Fuc) to a variety of acceptor molecules.^1^ These enzymes are essential in numerous biological processes, including protein glycosylation, cell signaling, immune response modulation, and pathogen recognition.^2^ Understanding these enzymes at a mechanistic level is crucial, as elucidating their catalytic pathways might enable the rational design of inhibitors for therapeutic purposes, supporting drug development strategies for diseases in which fucosylation plays a pivotal role.^3^

In particular, α1,6-fucosyltransferase (FUT8) is the sole mammalian enzyme responsible for catalyzing core α1,6-fucosylation of *N*-linked glycans. This reaction involves the transfer of an l-fucose residue from GDP-Fuc to the innermost *N*-acetylglucosamine (GlcNAc) of the chitobiose core, forming an α1,6-linkage, a process that is crucial for the maturation and function of glycoproteins (Figure 1).^4^

**Figure 1:**
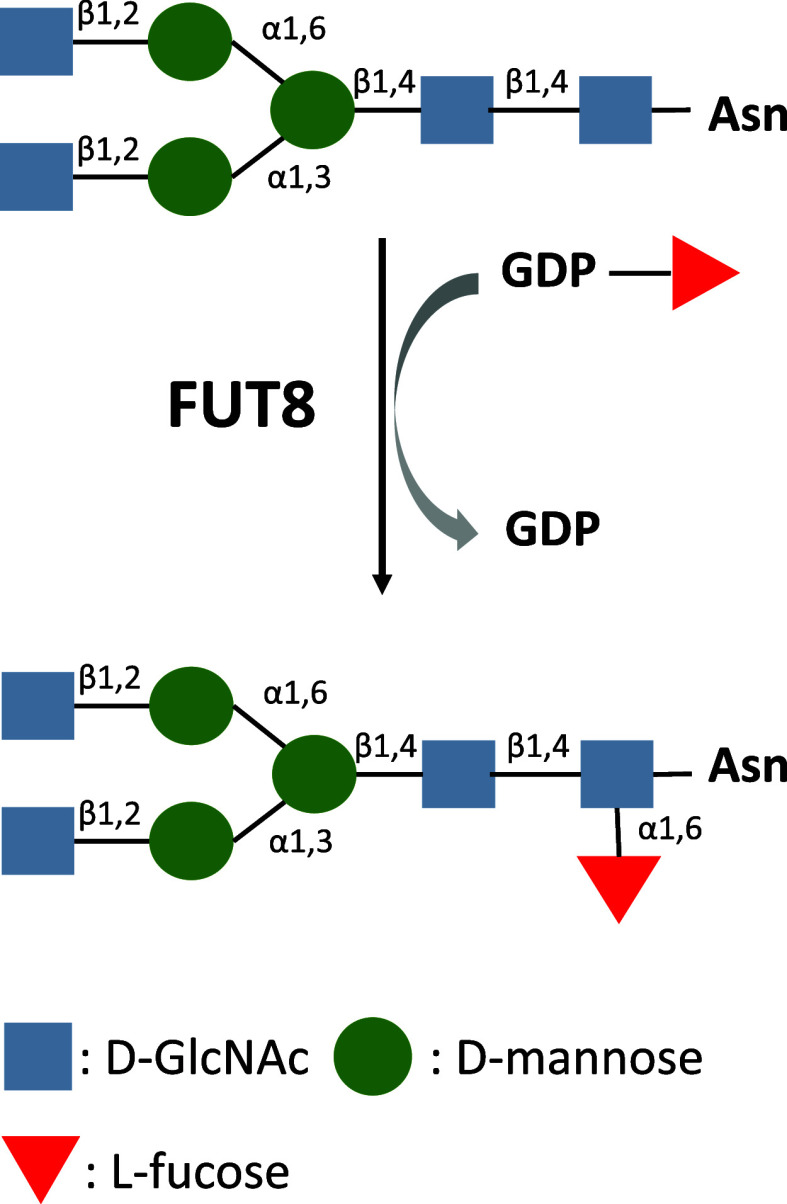
Reaction catalyzed by FUT8.

FUT8-mediated core fucosylation affects the activity of various glycoproteins, including transforming growth factor-β (TGF-β) receptors influencing diverse physiological and pathological processes.^5^ For instance, dysregulation of FUT8 has been implicated in cancer progression,^6^ inflammation,^7^ and mental diseases.^8^ making it a promising target for therapeutic intervention.^9^

Initial structural studies of mammalian FUT8 revealed a three-domain architecture comprising an *N*-terminal coiled-coil domain, a GT-B fold catalytic domain, and a C-terminal SH3 domain.^10^ The *N*-terminal coiled-coil domain mediates dimerization, which has been proposed to stabilize the enzyme and may be important for its catalytic efficiency.^11^ Subsequent high-resolution structures of human and mouse FUT8 bound to donor substrate analogues or GDP and various glycan acceptors further elucidated the molecular basis of substrate recognition and catalysis.^12^ These studies demonstrated that GDP-Fuc binds within a cleft formed by the two Rossmann-like domains of the GT-B fold, stabilized by conserved residues across vertebrate FUT8 homologues.^12b^ Recognition of the *N*-glycan acceptor by FUT8 involves coordinated interactions mediated by the GT-B fold, flexible loops, and the C-terminal SH3 domain, which specifically engages the α1,3-arm of the glycan.^12^ In particular, “loop 1” (residues 428–444), which is formed by a loop region and the adjacent α10 helix, and loop 2 (residues 365–378), corresponding to the β6−α8 loop, undergo substrate-induced concerted movements that enable active site closure, a process essential for catalysis. Loop movement to achieve active conformations in glycosyltransferases (GTs) is well-known, and there are several examples for the GT-A and GT-B families. However, while for GTs containing the GT-A fold the loop movement to reach active conformations has been computationally studied in some cases,^10^ taking advantage of the fact that only minor conformational changes are needed to form the Michaelis complex, multiple GT-B proteins (both retaining and inverting GTs) having been subjected to MD simulations (from ns to microsecond time scales, with and without ligand using a variety of force fields) which also reveal interconversion between the apo and liganded states/conformations.^13^ Crystallographic studies also proposed that FUT8 follows an S_N_2 inverting mechanism, in which Glu373 acts as the catalytic base. Structural comparison between the apo and ligand-bound form indicated that loop 2 repositioned Glu373 into the active site, enabling its function as the catalytic base, while loop 1 contributed to stabilizing the closed conformation (CC) required for proper substrate alignment and catalysis. In this mechanism, Glu373 is proposed to abstract a proton from the hydroxyl group of the acceptor glycan, thereby activating it for nucleophilic attack on the anomeric carbon of GDP-Fuc and enabling fucose transfer. Yet, the structural data alone could not resolve how substrate binding induces loop rearrangements, nor how these conformational dynamics are mechanistically linked to catalysis.

Here, we combine multiscale simulations to reconstruct the complete catalytic cycle of FUT8, integrating molecular dynamics, QM/MM calculations, and metadynamics. Our results reveal a previously unrecognized coordination between two dynamic loops, triggered by substrate binding, that drives active site assembly and enables fucose transfer through a slightly late S_N_2 mechanism with post-transition state proton transfer. This work connects substrate recognition, loop dynamics, and catalysis in FUT8 into a unified mechanistic model, representing, to our knowledge, the first study to employ this combined computational approach to resolve the full catalytic mechanism of a GT-B glycosyltransferase.

## Results and Discussion

### GDP-Fuc Binding Triggers Coordinated Loop Closure in FUT8

Molecular dynamics simulations were initiated from the crystallographic structure of FUT8-G0-GDP (PDB ID: 6TKV).^12a^ From this structure, a Michaelis complex (MC) model was derived by replacing GDP with GDP-Fuc. The simulations revealed distinct conformations of loops 1 and 2 when compared to the apo form (PDB ID: 2DE0).^10^ In the apo structure, the catalytic base Glu373 remained distant from the GDP-Fuc binding site. In the MC, however, a conformational rearrangement of loop 2 brought Glu373 into close proximity to the reaction center. These results are consistent with previous structural observations (Figure 2a).

**Figure 2:**
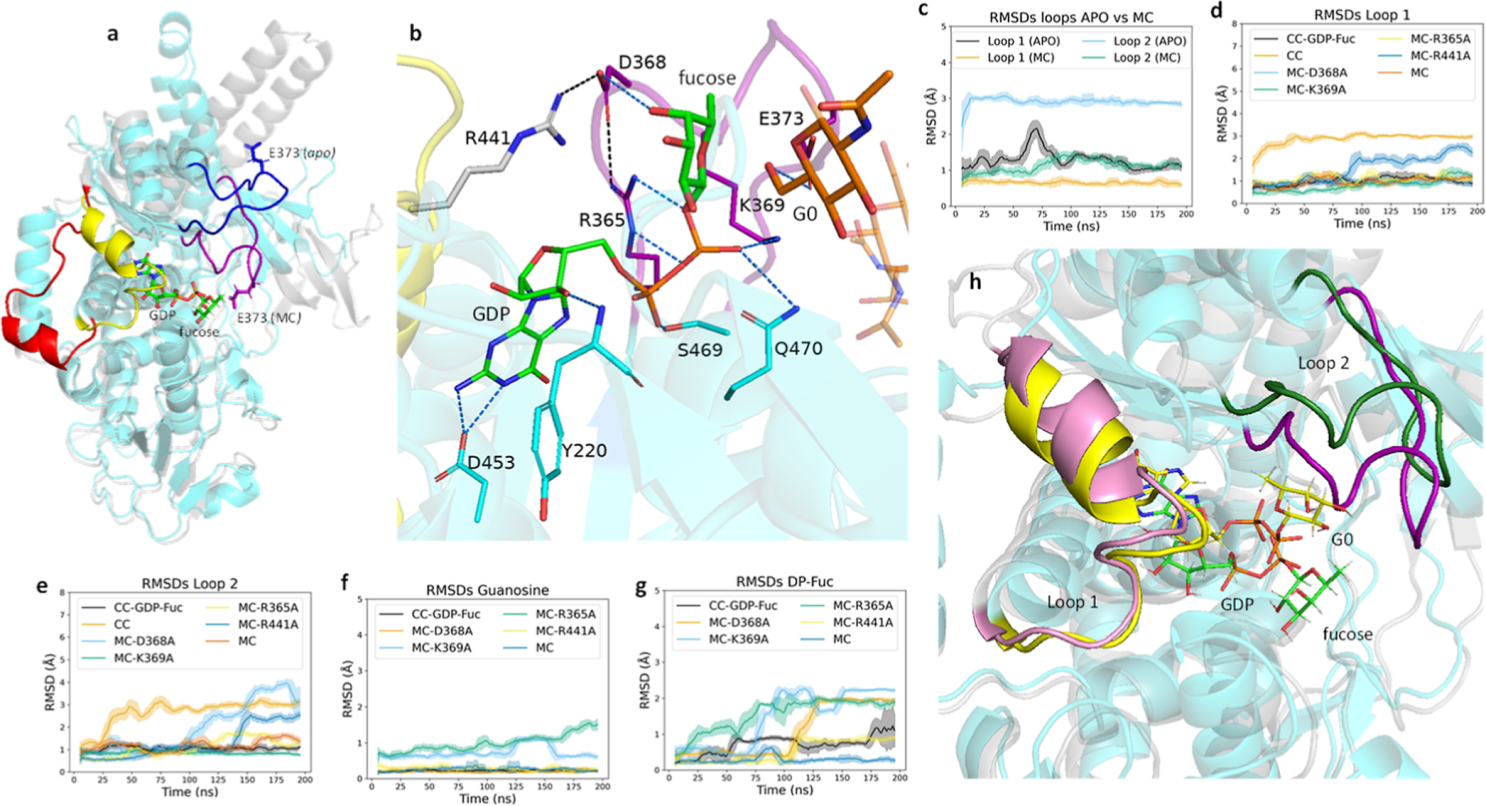
(a) Structure of apo FUT8 (gray) superimposed with the Michaelis complex (cyan) formed by FUT8 in the active form, GDP-Fuc and G0. Loop1 and loop2 are colored in red and blue for the apo form and in yellow and purple, respectively, for the Michaelis complex. GDP-Fuc (green) as oriented in the Michaelis complex and the catalytic base, Glu373, are shown as sticks. (b) Michaelis complex (cyan) showing H-bonding scheme (as dashed lines) in the active site. Interactions with GDP-Fuc (green) are shown in blue; interactions between Arg365, Asp368 and Arg441, responsible of the coupling between loops, are shown in black. Loop1 and loop2 are colored in yellow and purple, respectively. Substrate G0 (only partially showed) is shown in orange. (c–g) Root mean square deviation (RMSD) analysis of loop 1 and loop 2 across multiple simulations, comparing different FUT8 mutants (R365A, D368A, R441A, K369A) and complexes (MC, CC in complex with GDP-Fuc and CC in the absence of ligands). Shadows of lines represents the STDS which refers to the standard deviations computed across the five replicate simulations. (f,g) RMSD analyses of guanosine and DP-Fuc under different simulation conditions, showing increased DP-Fuc’s fluctuations in the mutants compared to the MC. STDS refers to the standard deviations computed across the five replicate simulations. (h) Representative snapshot illustrating the displacement of GDP-Fuc within the active site, with GDP-Fuc in the Michaelis complex (MC) shown in green and in the D368A mutant shown in yellow. Michaelis complex is oriented in the same way that in (a).

Those simulations also revealed that GDP-Fuc is stabilized through interactions with multiple residues in the active site (Figure 2b). Among these interactions, highly conserved contacts were observed with Arg365 and Lys369, both located in loop 2. Additionally, these simulations indicated that both loops remained structurally stable within this complex (RMSD <2), compared to the increased flexibility observed in loop 2 in its apo state (Figure 2c). Time-dependent distance analyses and principal component analysis (PCA) further support the proposed loop dynamics (see Supporting Information). The PCA results reveal that loop motions are significantly more restricted in the Michaelis complex compared to the apo enzyme (see Supporting Information). The apo form samples a broader conformational space along PC1 and PC2, while the Michaelis complex occupies a compact region, indicating loop stabilization upon substrate binding. The compact distribution along PC1 and PC2 and the lower variance values demonstrate that loop 2 becomes more rigid upon substrate binding, consistent with its role in stabilizing the catalytic conformation. The variance analysis confirms that these first two components capture the dominant collective motions of the loops. A highly conserved interaction was also identified between Arg441 (loop 1) and Asp368 (loop 2), which further interacts with Arg365 (loop 2), forming a three–hydrogen bond network. This interaction may play a crucial role in stabilizing the active conformation of FUT8.^12c^ Asp368 formed a hydrogen bond with the OH4 of fucose, remaining relatively stable during the first 100 ns of simulation (See Supporting Information). This suggests its role in properly orienting fucose for the transfer reaction (Figures 2b and S2)

To assess the impact of the ligands on active conformation stability, MD simulations of MC and the closed conformation (CC) without any ligand and in complex with GDP-Fuc, were carried out. These simulations emphasized the essential role of GDP-Fuc in stabilizing loop 2, which, in turn, contributes to loop 1 stability through the Asp368-Arg441 interaction. While G0 does not appear to contribute to the overall stability of CC, it facilitates the correct positioning of the Glu373 side chain. To evaluate the role of key residues in loop stability and ligand interactions, MD simulations were performed using the R365A, D368A, R441A, and K369A mutants, based on MC. The essential role of these residues in catalytic activity was demonstrated in previous experimental studies;^10,12b,14^ however, their specific functional contributions remain unclear. The results also indicated that the closed conformation was affected in the R441A and D368A mutants, as well as in ligand-free simulations, and in some R365A replicates (Figure 2d,e). The loss of stability in the R441A and D368A mutants highlights the key role of loop–loop interactions in maintaining the closed conformation. The increased RMSD of loops in some R365A replicates may be due to its role in Asp368 orientation, which is crucial for stabilizing loop–loop interactions.

The R365A and K369A mutants caused significant conformational instability in the diphosphate–fucose (DP-Fuc) region of GDP-Fuc, while the guanine moiety remained stably positioned throughout all simulations (Figure 2f,g). In both mutants, the altered orientation of GDP-Fuc underscores the essential role of Arg365 and Lys369 in donor substrate recognition and binding. On the other hand, in the R441A and D368A mutants, interactions between GDP-Fuc and loop 2 residues were maintained throughout all simulations, even when loop 2 opened (see Supporting Information). As a result, GDP-Fuc was observed to shift within the active site (Figure 2h). These findings suggest that GDP-Fuc exhibits a high affinity for residues Arg365 and Lys369 in loop 2, and that the proper orientation of these residues promotes the positioning of Asp368, enabling its interaction with Arg441 in loop 1. This network of interactions stabilizes the closed conformation of both loops and, consequently, the catalytically active state of FUT8. Considering the higher intrinsic flexibility of loop 2 observed in the apo form (Figure 2c), it is reasonable to propose that the loop closure process begins with the recognition of GDP-Fuc by loop 2. This initial event not only stabilizes the ligand itself but also acts as a structural trigger that facilitates closure of loop 1, thereby completing the transition to the enzyme’s active conformation. Notably, experimental binding data from previous studies also support this sequence of events. Isothermal titration calorimetry (ITC) showed that GDP binds to FUT8 with significantly higher affinity than the acceptor substrate G0, and that G0 binding is enhanced in the presence of GDP.^15^ This is fully consistent with our MD simulations, which reveal that GDP-Fuc stabilizes loop 2 and triggers the loop rearrangements required for catalytic activation. Together, these data corroborate a sequential binding model in which donor substrate binding precedes acceptor engagement, priming the active site for catalysis.

To investigate the completion of the catalytic cycle, MD simulations were performed on the reaction product complex, i.e, CC in complex with fucosylated G0 and protonated GDP which is obtained as a consequence of the proton-transfer assisted by Lys369 (see below), required after the reaction, to recover the catalytic base Glu373. The spontaneous release of G0-Fuc was observed (Figure 3a) with the concomitant shift of the catalytic base, giving rise to the new CC in complex with protonated GDP. Simulations of this complex showed increasing of GDP mobility within the active site, destabilizing the Arg365-Asp368-Arg441 interaction and facilitating loop opening (Figure 3b). Although the release of protonated GDP was not directly observed, it is reasonable to assume that, in the presence of excess GDP-Fuc, this ligand can replace the protonated GDP, thereby enabling the catalytic cycle to restart (Scheme 1).^16^

**Figure 3:**
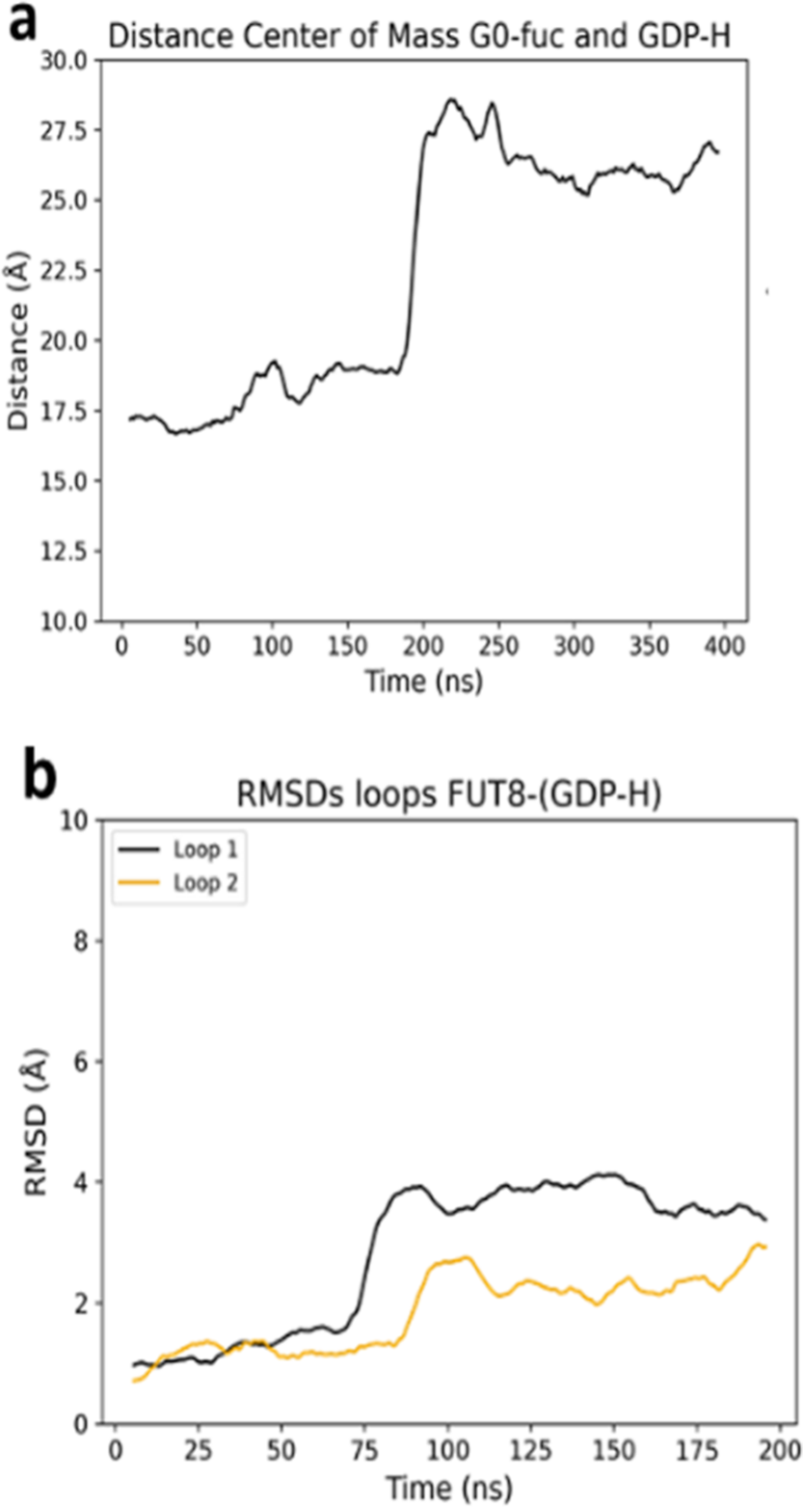
Fucose transfer completion leads to G0-Fuc release and loop opening in FUT8. (a) Spontaneous release of G0-Fuc from the active site into the solvent. (b) RMSD analysis of loop 1 and loop 2 in the FUT8-GDP complex.

**Scheme 1:**
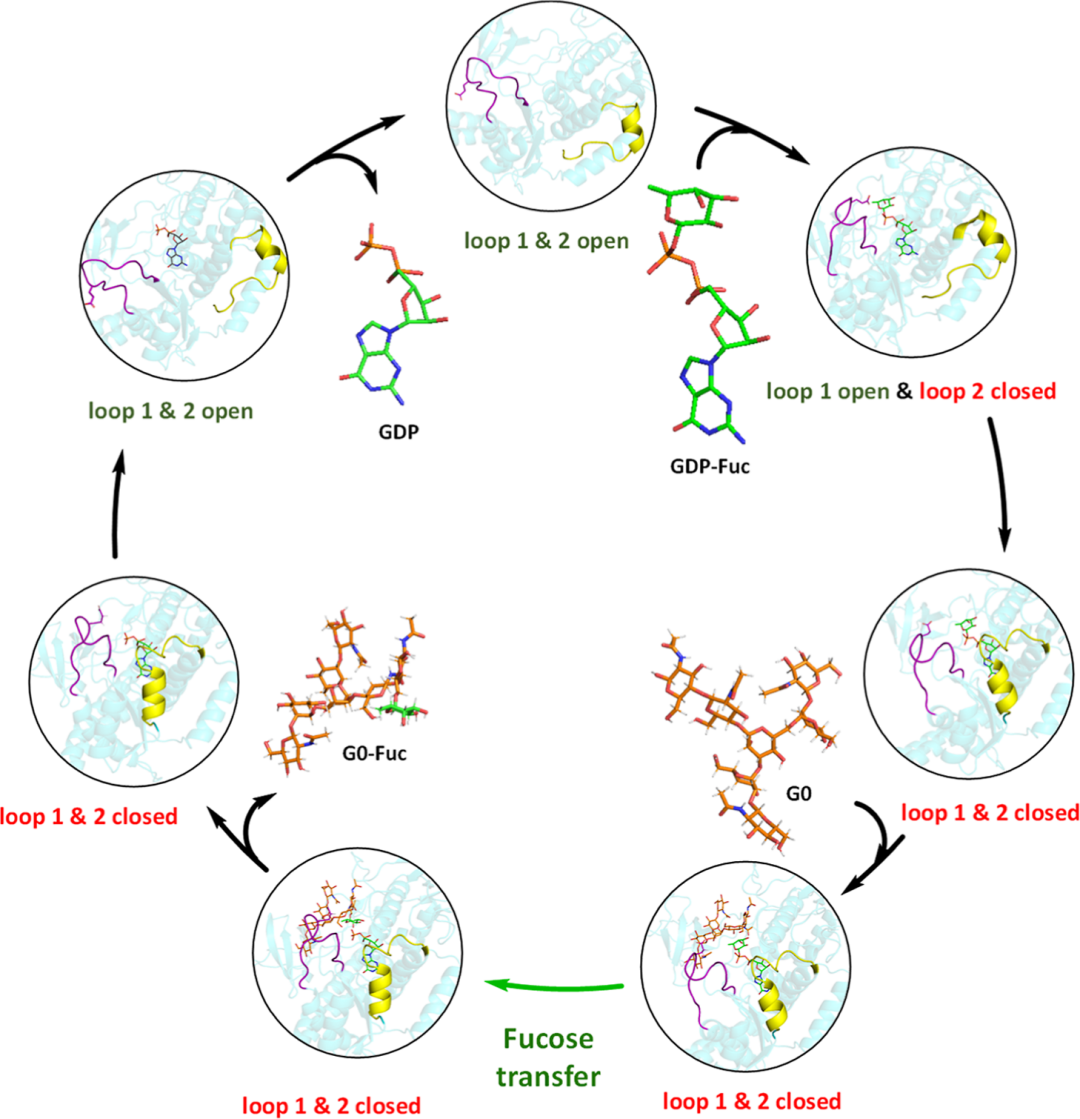
Catalytic Cycle of FUT8

a Schematic representation of the fut8 catalytic cycle, highlighting the structural transitions between open and closed conformations throughout the reaction process. In the figure, the protein is shown in cyan, with loop 1 in yellow, loop 2 in purple, GDP-fuc in green and G0 in orange. Notably, the intermediate state in which gdp-fuc is bound and loop 2 is closed was not directly sampled in the md simulations, but rather inferred from the observed stabilization patterns and the mutual interactions between GDP-fuc and loop 2 residues.

Altogether, these results support a model in which GDP-Fuc binding to loop 2 initiates a cascade of structural events that drive loop 1 closure and the formation of a catalytically competent active site. The interplay between Arg365, Asp368, and Arg441 acts as a conformational lock that maintains this closed state throughout catalysis. Upon product formation and G0-Fuc release, destabilization of this loop–loop interaction network permits active site reopening, enabling GDP exchange and completion of the catalytic cycle. This dynamic coordination between substrate recognition, loop rearrangement, and product release establishes a structural mechanism by which FUT8 regulates catalysis through conformational gating.

### A Slightly Late and Highly Asynchronous S_N_2 Mechanism Supported by QM/MM Calculations and Metadynamics

After identifying the key structural features governing the dynamics of the two loops in FUT8, a preliminary QM/MM study was performed to determine the key stationary points in the fucose transfer reaction (Scheme 2): the reactant (**RE**_**QM**_), transition state (**TS**_**QM**_), and reaction product (**PR**_**QM**_).

**Scheme 2:**
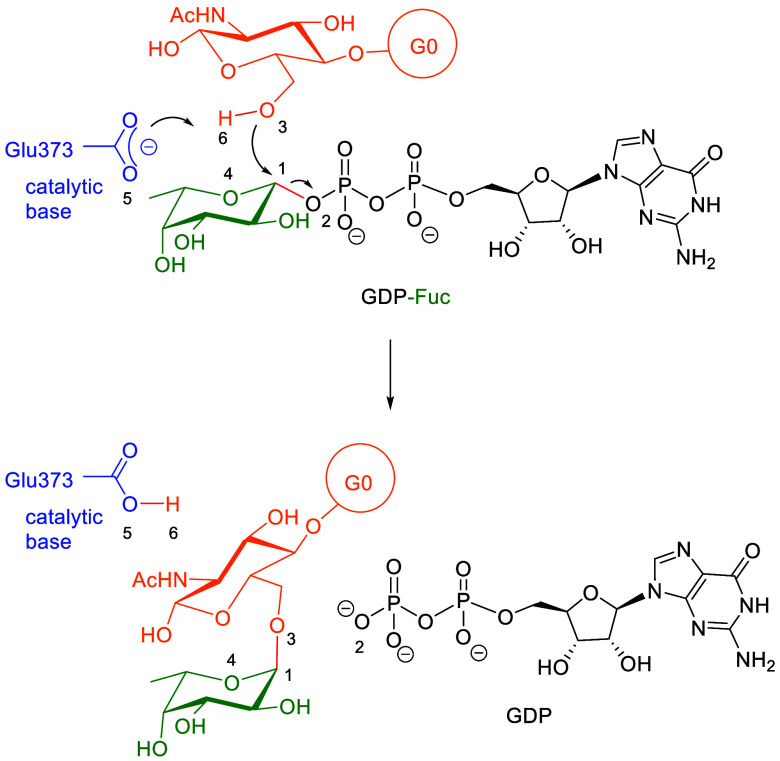
Reaction Corresponding to Fucose Transfer

The model chosen to study the reaction was obtained from the Michaelis complex analyzed above (For the full QM/MM study see Supporting Information). Key elements considered for the reaction included the catalytic base Glu373, along with the residues Arg365 and Lys369, which are necessary to form a salt bridge with the pyrophosphate acting as the leaving group.

The mechanism was expected to proceed like other inverting glycosyltransferases through an S_N_2 mechanism without the formation of intermediates. In this context, we have reported the mechanisms of other fucosyltransferases^17–19^ transferring the fucose unit to epidermal growth factor-like (EGF) repeats and thrombospondin type I repeats (TSRs), and in both cases, a pure S_N_2 mechanism was observed.

The geometry of the transition structure **TS**_**QM**_ was coherent with a late TS, as suggested from distances of involved bonds (2.0 and 2.3 Å for the forming and breaking bonds, respectively). These are in agreement with a S_N_2 reaction proceeding in a single step involving a ^3^H_4_ conformation for the fucose unit but with a high degree of asynchronicity. This high degree of asynchronicity between phosphate departure and nucleophilic attack supports a mechanism on the S_N_1-S_N_2 Frontier, in line with previous characterizations of some inverting GTs.^20^ However, to our knowledge, this is the first detailed computational characterization of such a mechanism in FUT8. The asynchronous nature of the transition state observed here also echoes mechanistic features previously described in certain retaining GTs,^21^ suggesting similar convergent catalytic strategies among GTs that differ in the stereochemistry of their products.

Metadynamics simulations were then carried out to unravel the complete fucose transfer process within catalytic itinerary of FUT8 (see Scheme 1) and to shed light on the possibility of an asynchronous S_N_2 reaction, as previously mentioned. These results indicate that the most stable conformation in the MC is ^1^C_4_ for fucose and ^4^C_1_ for the innermost GlcNAc (see Supporting Information). The free energy surface (FES) associated with the transfer reaction was designed using two collective variables (CVs): CV1 and CV2 where CV1 represents the fucose transfer process (CV1 = *d*_1_–*d*_2_) and CV2 describes proton transfer (CV2 = *d*_3_–*d*_4_) (Figure 4). Specifically, *d*_1_ is the distance between the anomeric carbon (C1) and the diphosphate oxygen (O2), *d*_2_ is the distance between C1 and the acceptor O3, *d*_3_ is the distance between O3 and its hydroxyl proton (H6), and *d*_4_ is the distance between the H6 and the Glu373 carboxylate oxygen (O5) (see Scheme 2 for numbering).

**Figure 4:**
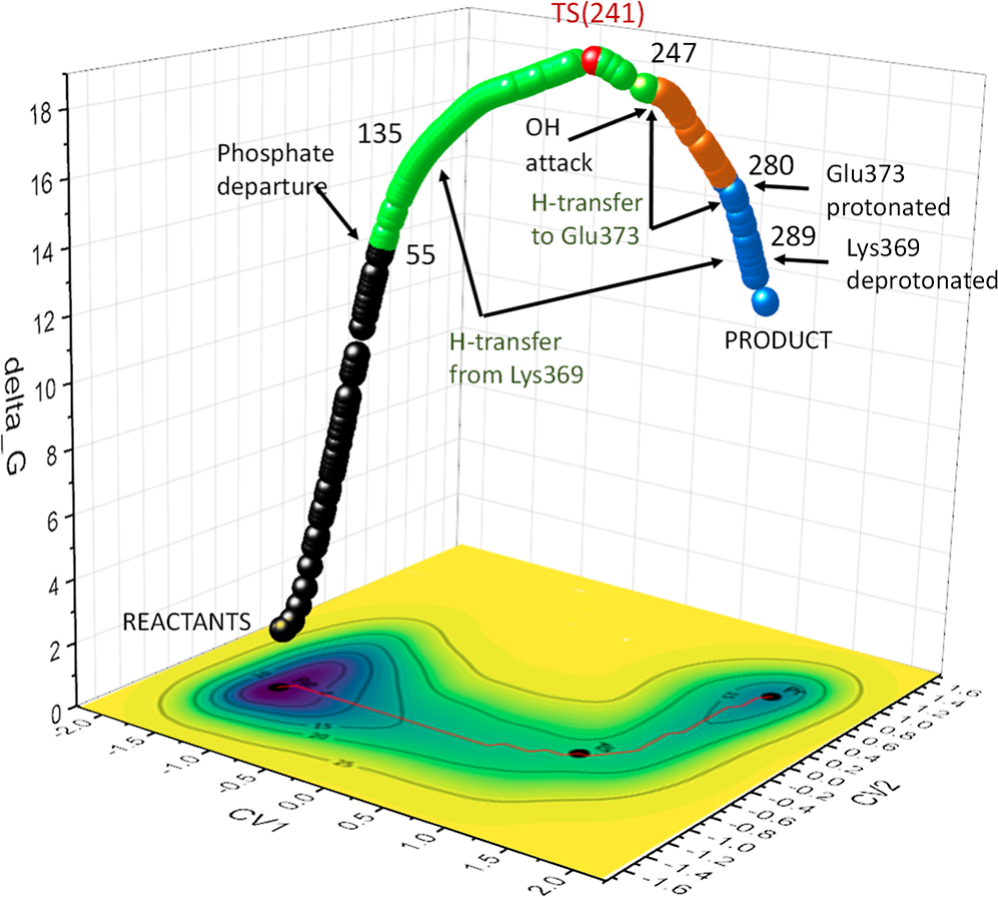
Free energy surface (FES) of the glycosylation reaction from metadynamic simulations. The reaction path (red in the contour map) connects RE and PR through TS. The 3D route is formed by 300 points extracted from the reaction map. The different stages of the reaction according to ELF analysis (see below) are diversely colored. Transition state is given in red. Numbers refers to the point of the reaction path.

In this case, it was also necessary to include the side chain of residue Gln470 within the QM region, to obtain a more accurate description of the FES in the transfer reaction. Each point on the FES (Figure 4) corresponds to a different stage of the reaction. The *X*-axis represents the progress of bond formation between the anomeric carbon C1 and the O6 of the innermost GlcNAc unit in G0, while the *Y*-axis represents the proton transfer, from O3 to Glu373. Two distinct minima are clearly identified, corresponding to the initial (**RE**_**meta**_) and final (**PR**_**meta**_), states of the reaction, along with the transition state (**TS**_**meta**_) (Figure 5a) with a free energy barrier of 18.0 kcal/mol, a value consistent with that observed in static QM/MM calculations (18.0–19.7 kcal/mol correspondig to *E*_electronic_). We did not include ZPVE contibution because it can only be acurately computed for the QM region. Vibrational modes involving atoms at the QM/MM boundary or in the MM region are not properly described at the quantum level, making ZPVE calculation incomplete or unreliable. The energy barriers calculated through metadynamics and QM/MM fall within the range reported in the kinetic studies published to date (see Supporting Informaton).

**Figure 5:**
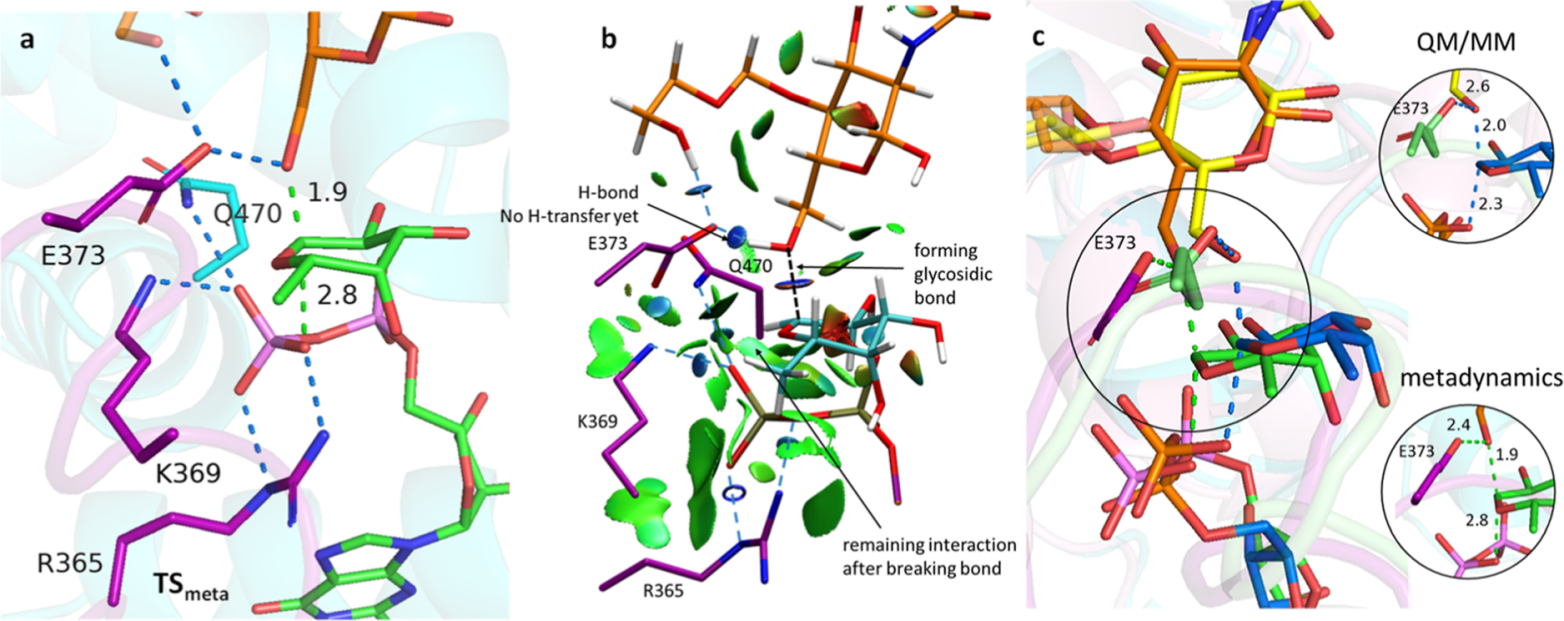
(a) Gometry of **TS**_**meta**_ showing H-bonds (blue) and breakin/forming bonds (green). (b) NCI calculations of **TS**_**meta**_. Thin, delocalized green surface indicates van der Waals interactions. Small, lenticular, bluish surfaces indicate strong interactions such as hydrogen bonding. Steric clashes are shown as red isosurfaces. The color code of fucose (now cyan) has been changed for clarityy. (c) Overlapping of **TS**_**QM**_ and **TS**_**meta**_, showing breaking and forming bonds. Hydrogens have been omitted for clarity.

A larger difference between forming and breaking bonds (1.9 Å and 2.8 Å for *d*_2_ and *d*_1_, respectively) was observed for **TS**_**meta**_ (Figure 5a) with respect to **TS**_**QM**_. The values observed for **TS**_**meta**_ suggested a much more asynchronous reaction in the borderline with a S_N_1 mechanism. Indeed, the calculated distances O5–H6 (1.5 Å) and H6–O3­(G0) (1.0 Å) confirm that the proton transfer takes place after the transition state, allowing it to be considered a late transition state. This situation was further confirmed by a NCI analysis,^22^ which also showed interactions of Arg365 and Lys369 (Figure 5b) were found to be very similar in both **TS**_**QM**_ and **TS**_**meta**_. On the other hand, while Gln470 has little influence in **TS**_**QM**_, some interactions with the pyrophosphate unit are found in **TS**_**meta**_ as also illustrated the NCI analysis. Interestingly, **TS**_**meta**_ showed additional interactions of Asp368 with the fucose unit. Despite these differences, an overlapping of both transition structures displays similar orientation and conformation for both fucose and GlcNAc units (Figure 5c).

The complete pathway can be obtained from the free energy surface (FES) of the metadynamic simulations. In order to gain a deeper understanding of the events occurring throughout the reaction, an analysis of the electron localization function (ELF)^23^ was carried out on 300 points extracted from the free energy surface (FES) along the minimum energy path between the two minima (Figure 4). ELF analysis allows the monitoring of the evolution of electronic density through so-called attractors, which represent local maxima of electron density in various spatial regions (referred to as basins). By tracking these maxima, it is possible to determine the moment a bond is broken, as this corresponds to a transition from a shared basin between two atoms (disynaptic) to two independent basins, each associated with a single atom (monosynaptic), or in some cases, just one basin if there is a significant electronegativity difference between the atoms. Bond formation follows the reverse pattern. Although ELF analysis has been successfully used for the in silico characterization of hidden intermediates and transient species,^24^ there are few precedents for the application of this type of topological calculations in enzymatic reactions.^25^

ELF analysis Figure 6; for the graphic showing the evolution of electron population see Figure S16 revealed that, although the reaction proceeds via a concerted S_N_2 mechanism with a single transition state, it occurs through several well-defined stages or events. The first event is the cleavage of the bond between the anomeric carbon and the phosphate group, which takes place at point 55 (Figure 6). Unlike what has been observed in other fucosyltransferases,^18^ the diphosphate moiety is minimally exposed to the solvent, requiring the presence of Gln470, Lys369, and Arg365 to promote the departure of the phosphate leaving group through the formation of H-bonds. The resulting species once the glycosidic bond with the phosphate is broken (Figure 6, point 56) is a transient glycosyl cation which, actually, could be considered an intimate ion pair. Although this species does not correspond to a classical intermediate, since it does not represent an energy minimum, it possesses sufficient character to be considered a distinct stage of the reaction, and thus qualifies as a transient carbocation.^26^ After some points (Figure 6, point 135) we observed the start of a H-transfer from Lys369 toward the pyrophosphate group (to a different atom from the one previously bonded to the anomeric carbon). This H-transfer, which had not been observed in QM/MM calculations, take place smoothly and it was not completed until point 289. The transient carbocationic species, which we have defined and experimentally demonstrated for typical organic reactions,^24a,24b,27^ having a well-defined geometry, persists until point 247, where the new glycosidic bond is formed; this constitutes the second event in the reaction, as observed through the ELF attractors. This situation is likely due to the requirement for a sufficient charge defect before the hydroxyl group, which has some but not strong nucleophilic character, can engage in the nucleophilic attack; notably, initial deprotonation by Glu373 does not occur at any point. Thus, once adequate charge transfer has developed, the nucleophilic attack by the hydroxyl group takes place at points 247–248. At that moment, following bond formation, the hydroxyl group acquires enough acidity to enable hydrogen transfer to the catalytic base Glu373, constituting the third event of the reaction, starting at point 251. The H-transfer from G(0) to Glu373 was completed at point 280. These observations do not in any way imply that the process is stepwise, which would lead to a glycosyl cation and, consequently, to an S_N_1-type reaction. The process is an asynchronous S_N_2-type reaction, in which the formation of the new glycosidic bond occurs after the transition state, almost simultaneously to the proton transfer from O3 to the catalytic base, Glu373, and accompanied from a second H-transfer from Lys369 to the pyrophosphate. Actually, these reactions move in a S_N_1/S_N_2 mechanistic continuum that depends on the time gap between leaving group departure and nucleophilic attack.^28^ In any case, there is no prior deprotonation of the hydroxyl group that attacks the anomeric carbon of fucose; the proton transfer to the catalytic base Glu373 is concomitant with the formation of the glycosidic bond. Hence, it is necessary for the C­(anomeric)-O­(phosphate) bond to break initially in order to generate a charge defect that serves as the driving force for the nucleophilic attack of O6 of GlcNAc. Moreover, Lys369 does not assist to the leaving of the pyrophosphate group since the H-transfer takes place after the anomeric bond when the pyrophosphate is broken. Indeed, this additional H-transfer contributes to the protonation of GDP facilitating its departure together with fucosylated G(0), the products of the reaction. After the transfer, deprotonated Lys369 remains in close proximity to protonated Glu373, which could facilitate an acid–base equilibrium process between these residues. This mechanism would allow the restoration of the original protonation state of FUT8 in its active form enabling it to continue with another catalytic cycle for the fucose transfer (Scheme 3) embedded within the whole catalytic cycle illustrated in Scheme 1.

**Figure 6:**
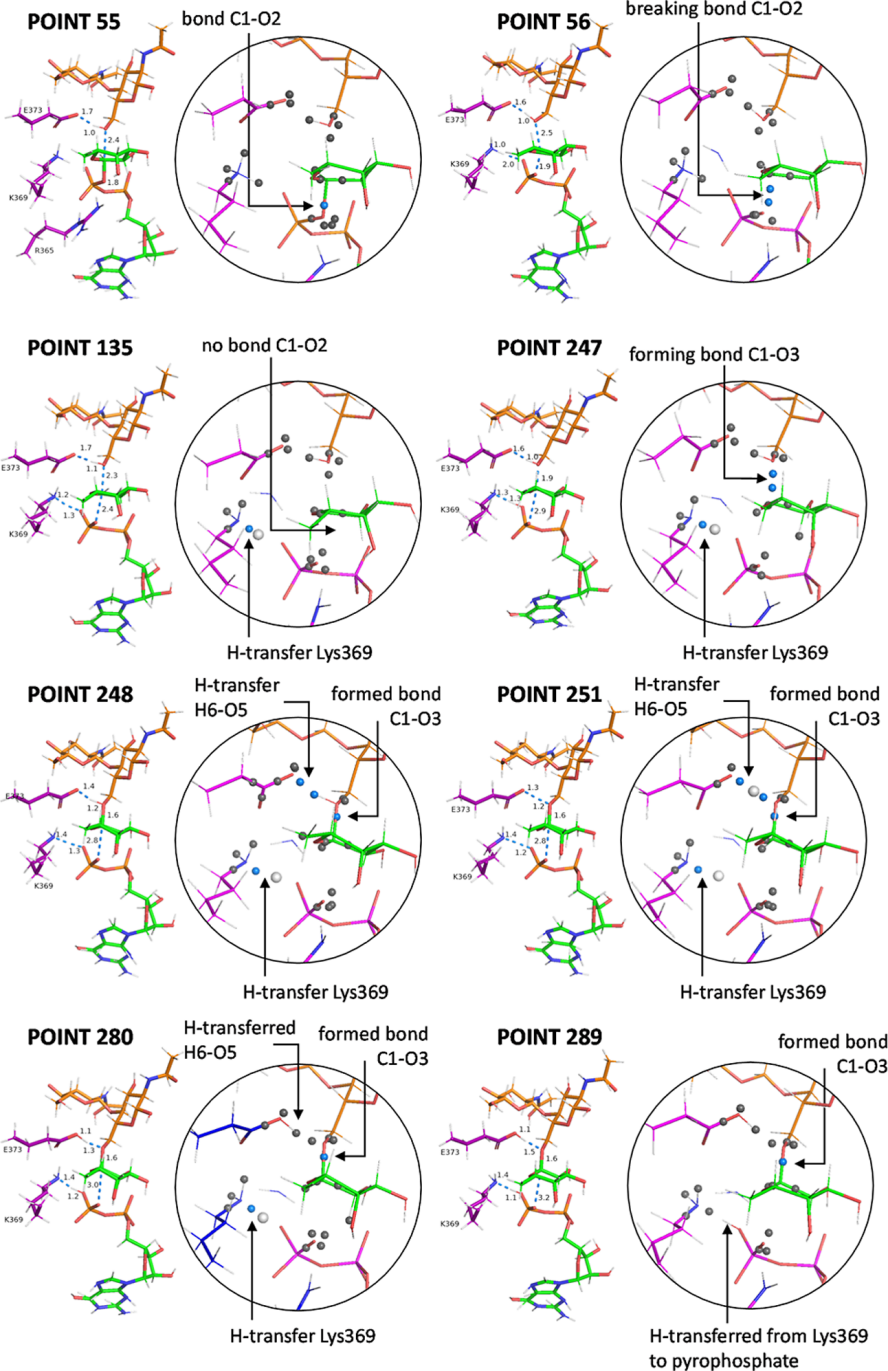
Sequence of snapshots corresponding to the different events occurring throughout the reaction pathway illustrated in Figure 5: (1) breaking of C1–O2 bond; (2) H-transfer from Lys369 to pyrophosphate; (3) formation of C1–O3 bond; (4) H-transfer of H6 from O3 to O5 of Glu373. Atom numbering corresponds to that given in Scheme 2. The dots represent the 300-point minimum energy path indicated in Figure 4. The black circle highlights the event shown in detail, and the most representative ELF attractors have been added as gray or blue spheres. The blue spheres correspond to the attractors involved in the event taking place in each snapshot. A sphere (attractor) located between two atoms indicates a bond; if two spheres subsequently appear in the same region associated with the bonded atoms (monosynaptic basins), the bond is being broken at that moment. The opposite process indicates bond formation. If spheres appear along a hydrogen bridge A–H····B, they indicate a hydrogen transfer event, which may last several femtoseconds. The larger white spheres that occasionally appear represent the hydrogen atom being transferred. G0 is colored orange, the amino acid residues involved are shown in magenta, and fucose is shown in green. The corresponding graphic showing the evolution of the electron population is given in Figure S16.

**Scheme 3:**
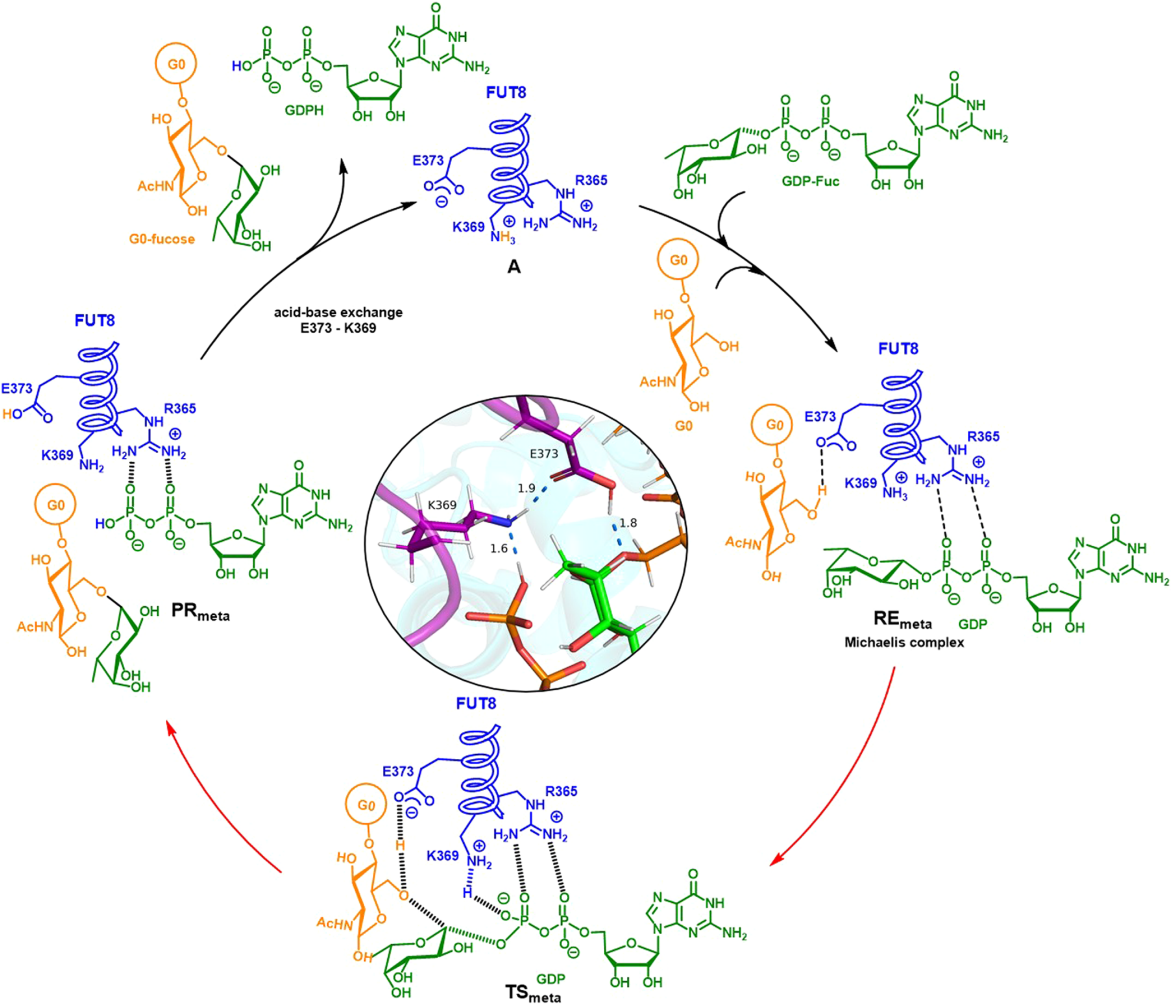
Proposed Cyclic Pathway for the Fucose Transfer Catalyzed for FUT8^a^

a In the center of the image there is a detail of the h-bond network as found in the immediate product **pr**_**meta**_. the reaction starts with the formation of the michaelis complex by incorporating gdp-fucose and g0 to the active site of the enzyme. then the reaction takes place, that is the leaving of the pyrophosphate unit, the nucleophilic atttack of o3 of g0 to the anomeric carbon of the fucose and finally de H-transfer from O3–H6 to the cataytic base. In the last step, acid-base exchange with the solvent equilibrate the system and the catalytic base was deprotonaded and the pyrophosphate Protonated

Noteworthy, that second H-transfer was not detected by QM/MM calculations and it leads to an immediate product that requires the assistance of an external solvent to recover the protonation state. In the case of QM/MM/MD calculations, the immediate product is simply the enzyme, effectively in its initial state, since we are only considering the position of a hydrogen atom between the donor and acceptor atoms which, in fact, represents a single unique state.

All these stages are represented along the minimum energy path (see Scheme 1), which corresponds to the set of points from which the ELF analysis was typically performed (Figure 6). That ELF analysis can be considered analogous to a conventional IRC analysis in QM calculations. However, it provides significantly more detailed and accurate information than a simple bond-breaking/forming diagram, as it monitors the evolution of the electron density. This allows us to determine the exact moment a bond is broken or formed. Both representations, however, unfold along the reaction coordinate and not over time. To access time-resolved information, a MD trajectory must be defined. Due to the inherently stochastic nature of these simulations, multiple replicas must be performed. In this context, and in order to extract information about the average lifetime of the transient carbocation, a set of 20 QM/MM/MD simulations was carried out over 4 ps, starting from the transition state structure obtained through metadynamics. To ensure that the two half-trajectories formed a complete trajectory from the reactant to the product passing through the transition state, they were launched in pairs with the same velocity but in opposite directions, while maintaining in all cases the stochastic component of a molecular dynamics simulation. (for details see Supporting Information). Subsequently, ELF analysis was performed for each trajectory. The ELF-MD analyses for the 20 simulations are depicted in Figure 7 (only those basins corresponding to selected atoms and bonds). The data presented in Figure 7 represents the evolution of the electron population during the reaction of glycosylation, but in this case, over time, unlike when it is done using the reaction coordinate data, providing a much more realistic view of how the reaction takes place.

**Figure 7:**
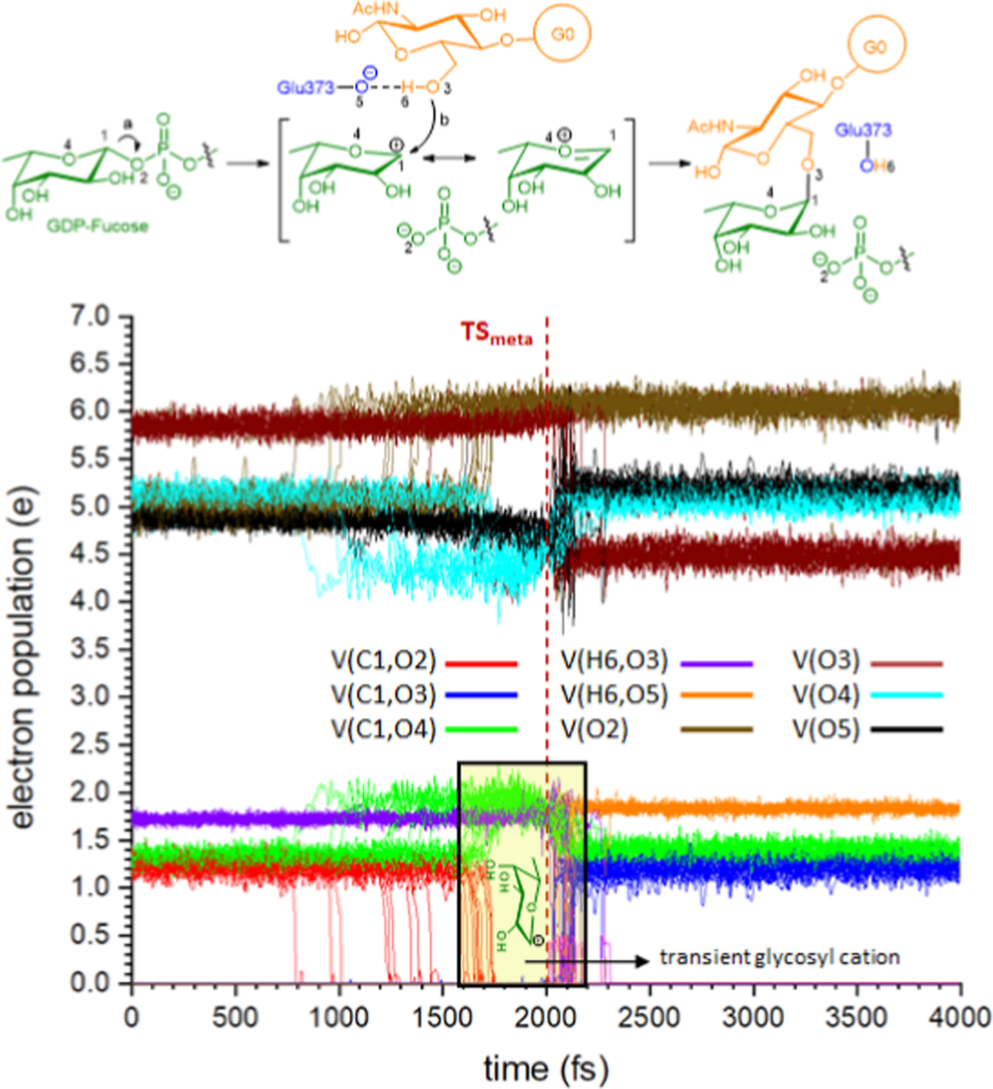
ELF-MD analyses of the QM/MM/MD simulations carried out starting from **TS**_**meta**_ during 4 ps. Both trajectories leading to **RE**_**meta**_ and **PR**_**meta**_ are depicted, the former in reverse pathway (for the individual trajectories see Supporting Information). V­(*x*,*y*) corresponds to the population of the bond between X and Y. V­(*x*) corresponds to the population of lone pairs of X. It can be seen (a) in the reaction scheme that when the bond C1–O2 is broken O2 increase its density as a consequence of receiving that from the bond. When C1–O3 is formed (b) in the reaction scheme, O3 fluctuates as a consequence of the influence and concomitant H-transfer to Glu373. In the middle it is observed some increase of density for the bond C1–O4 supporting the stabilization by resonance of the glycosyl cation.

The data presented in Figure 7 clearly show that there is a time interval between the cleavage of the bond with pyrophosphate and the formation of the new glycosidic bond. During this interval, an increase in the population of the bond between the anomeric carbon and the endocyclic oxygen is observed, suggesting the presence of a glycosyl cation stabilized by resonance. The other atoms and bonds evolve accordingly.

Admittedly, the stochastic nature of molecular dynamics simulations requires performing a certain number of replicas to assess reproducibility. In fact, we can observe gaps up to 800 fs but in any case, less than 300 fs. Although not extremely precise, this methodology allows, for the first time, the evaluation of a transient situation corresponding to a transient carbocation, in such a way that a lifetime can be assigned in our case, between 300 and 800 fs. Therefore, we cannot confirm a genuine S_N_1-like process with inversion of configuration (as typically observed for an intimate ion pair). Instead, we consider it more accurate to describe the process as an S_N_2 reaction at the Frontier with an S_N_1 mechanism, still leading to inversion of configuration.

## Conclusion

This study elucidates FUT8’s catalytic cycle by integrating insights into loop dynamics and reaction mechanisms through MD simulations, QM/MM calculations, and metadynamics. GDP-Fuc binding stabilizes loop 2 in a closed conformation, which in turn enables its interaction with loop 1. This interloop coupling, mediated by a conserved hydrogen bond network (Arg365–Asp368–Arg441), locks FUT8 into its catalytically active state. The resulting architecture precisely orients both GDP-Fuc and the catalytic Glu373, priming the enzyme for fucose transfer upon G0 binding. Mutational studies confirm that disrupting this network destabilizes both, loops and GDP-Fuc, impairing catalysis. After fucose transfer, G0-Fuc departure weakens GDP interactions, disrupting the hydrogen bond network and triggering loop reopening, thereby resetting the enzyme for the next catalytic cycle.

Crucially, QM/MM and, particularly, metadynamics simulations reveal that FUT8 does not follow the canonical S_N_2 mechanism typically associated with inverting glycosyltransferases. Instead, it proceeds via an asynchronous S_N_2 mechanism, wherein cleavage of the glycosidic bond between fucose and GDP occurs first, followed by a coupled glycosidic bond formation and proton transfer to Glu373. These events take place along a single reaction coordinate without formation of a stable intermediate, and through a late transition state, with an overall energy barrier (∼18 kcal/mol) consistent with experimental *k*_cat_ values. To our knowledge, this represents a clear example of a glycosylation reaction at the S_N_1-S_N_2 interface^21,24b,29^ as it is, actually, a highly asynchronous nucleophilic substitution with an only transition state. Moreover, the application, for the first time, of topological analysis to the entire course of an enzymatic reaction has made it possible to determine the existence of a transient glycosyl cation lasting between 350 and 800 fs. This is a rare example for an inverting glycosyltransferase of which there are very few examples,^30^ introducing a mechanistic variant previously described only in selected enzymatic systems such as phosphoryl^31^ or methyl transfer.^31b^ The combination of ELF analysis with QM/MM/MD simulations results in a new protocol for accurately understanding how the reaction takes place, as it shows at what moment, on a time scale, a bond is formed or broken, or an atom gains or loses electronic density.

Throughout catalysis, GDP-Fuc remains coordinated through interactions with Arg365, Lys369, and Gln470, while Asp368 guides fucose positioning.^32^ In addition, metadynamics simulations identify a spontaneous proton transfer from Lys369 to GDP, suggesting its role in stabilizing the charged reaction product and restoring the system’s protonation balance. A study of the evolution of the electron density along the reaction as defined by metadynamics simulations supports the formation of an intimate ion pair which, although it does not correspond to a local energy minimum, can be identified as a distinct species throughout the course of the reaction. Taken together, our findings define a mechanistically distinct catalytic paradigm in GT-B enzymes, directly linking ligand-induced conformational dynamics with chemical transformation, and offering a framework for designing transition-state inhibitors targeting core fucosylation in disease contexts such as cancer.

## General Methods

### Molecular Dynamics Simulations

All molecular dynamics (MD) simulations were conducted using the AMBER20 software suite. Protein parameters were assigned using the ff14SB force field, while carbohydrate components were treated with the GLYCAM06j force field. Small organic molecules and ligands were parametrized using the general AMBER force field (GAFF). Partial atomic charges were computed using the antechamber module with the AM1-BCC method. In cases requiring higher accuracy, RESP charges were derived from HF/6–31G* electrostatic potentials computed with Gaussian, followed by fitting using AMBER’s resp utility. System building and solvation were performed with tleap. Each system was solvated in an octahedral box of TIP3P water molecules extending at least 12 Å from any solute atom. Counterions (Na^+^) were added to neutralize the system. Energy minimization was carried out in two stages using sander: a restrained minimization with positional restraints (10 kcal/mol·Å^2^) on solute heavy atoms, followed by an unrestrained minimization of the entire system. Equilibration was performed under NPT conditions (1 atm) for 500 ps using a Monte Carlo barostat. Production MD simulations were run in the *NPT* ensemble using r pmemd.cuda (for GPU-accelerated runs), with a time step of 1 fs. All bonds involving hydrogen atoms were constrained using the SHAKE algorithm. Temperature was regulated via Langevin dynamics with a collision frequency of 1 ps^–1^. Long-range electrostatics were treated using the Particle Mesh Ewald (PME) method, and a 10 Å cutoff was applied to nonbonded interactions.

Trajectory analysis was performed using cpptraj, including RMSD, hydrogen bonding, and clustering analyses. Snapshots were extracted at regular intervals for structural and energetic evaluation.

### QM/MM Calculations

QM/MM calculations were performed using the ChemShell package,^33^ which provides an hybrid framework for combining quantum mechanics (QM) and molecular mechanics (MM). The QM region was treated with Gaussian09 ^34^ as the external quantum engine, employing the density functional theory (DFT) method wb97xd with the def2svp basis set, unless otherwise stated. The MM region was modeled using the AMBER force field, as implemented via the DL_POLY interface in ChemShell.

The QM/MM boundary was handled using hydrogen link atoms to cap covalent bonds crossing the QM/MM interface. Electrostatic embedding was employed, allowing the MM point charges to polarize the QM electronic density. Geometry optimizations were performed using ChemShell’s internal optimizer, acting on the QM region and selected flexible residues from the MM environment (typically within a 12 Å radius of the active site). The rest of the system was kept fixed to reduce computational cost.

Initial structures were prepared from equilibrated classical MD trajectories using AMBER. Representative snapshots were extracted and converted into ChemShell-compatible input using the pdb 2g94 and amber2chm utilities. Transition states were located using the dimer method or constrained optimization methods within the ChemShell framework. Frequency calculations were performed in Gaussian09 to confirm the nature of stationary points (one imaginary frequency for TSs; none for minima). Single-point energy refinements were optionally performed at different levels (see Supporting Information).

### QM/MM/MD and Metadynamics Calculations

QM/MM metadynamics simulations were performed to analyze the conformational behavior of the two sugars involved in fucose transfer within the Michaelis complex (MC), to model the reaction mechanism, and to determine the free energy surface of the glycosylation reaction. For this purpose, the CP2K software^35^ was used in combination with the metadynamics algorithm implemented in PLUMED 2.^36^ All metadynamics simulations followed a standardized protocol: (i) a multistep annealing process. (ii) a QM/MM simulation without an external potential at 300 K in an *NVT* ensemble for 5 ps, using a time step of 0.5 fs, to equilibrate the system at the QM/MM level and (iii) the metadynamics simulations using as the starting point the final snapshot from the equilibration. The metadynamics simulations were performed using the standard (non–well-tempered) algorithm as implemented in **PLUMED2.** This choice was motivated by the relatively simple reaction coordinate and the moderate height of the free-energy barriers involved, for which standard metadynamics provides sufficiently smooth and converged sampling. Gaussian potentials were deposited every 100 MD steps with an initial height of 1.0 kcal·mol^–1^ and a width of 0.2 for each collective variable (CV). To ensure numerical stability near the transition state, the Gaussian height was gradually reduced to 0.1 kcal·mol^–1^.To explore the reaction pathway from the transition state structure, we performed a set of molecular dynamics simulations using the CP2K package. Each simulation was initiated from the optimized transition state geometry, and atomic velocities were assigned according to the Maxwell–Boltzmann distribution at 298 K. To probe both directions of the reaction coordinate, we implemented a pairwise velocity scheme: for each simulation with an initial velocity vector *v*, a corresponding simulation was launched with inverted velocities −*v*, preserving the same initial positions. This approach ensures that the system evolves forward and backward along the reaction coordinate from the transition state (**TS**_**meta**_), enabling the generation of bidirectional trajectories that either lead to reactants or products.

A single initial velocity file was generated and then creating its inverted counterpart by multiplying all velocity components by −1. This can be done manually editing the velocity file. (we used in-house scripts) Both sets were used as input for independent MD runs under the same simulation conditions (ensemble, time step, thermostat, etc.).

All trajectories were propagated in the *NVE* ensemble (or *NVT* if temperature control was required), with a time step of 0.5 fs and a total simulation time of 200 ps per trajectory. The resulting data enabled time-resolved analysis of electronic structure descriptors, such as the electron localization function (ELF), along physically meaningful dynamical paths.

## Supplementary Material







## Notes

The following files are available free of charge (PDF)
Computational methods; additional figures; discussion on QM/MM calculations, details of ELF analyses, movies of the reaction and the evolution of descriptors (MP4)
descriptors-ELF.mp4; Reaction.mp4 (MP4)

## Abbreviations

CC: closed conformation
CV: collective variables
ELF: electron localization function
FES: free energy surface
FUT8: α1,6-Fucosyltransferase 8
MC: Michaelis complex
NCI: noncovalent interactions
PES: potential energy surface

## References

[1] Schneider M., Al-Shareffi E., Haltiwanger R. S. (2017). Biological functions of fucose in
mammals. Glycobiology.

[2] Becker D. J., Lowe J. B. (2003). Fucose: biosynthesis and biological function in mammals. Glycobiology.

[3] Pijnenborg J. F. A., Rossing E., Merx J., Noga M. J., Titulaer W. H. C., Eerden N., Veizaj R., White P. B., Lefeber D. J., Boltje T. J. (2021). Fluorinated rhamnosides
inhibit cellular fucosylation. Nat. Commun..

[4] Pan Q., Zhang X. L. (2025). Roles of core fucosylation
modification
in immune system and diseases. Cell Insight.

[5] Tu C. F., Wu M. Y., Lin Y. C., Kannagi R., Yang R. B. (2017). FUT8 promotes
breast cancer cell invasiveness by remodeling TGF-β receptor
core fucosylation. Breast Cancer Res..

[6] Osumi D., Takahashi M., Miyoshi E., Yokoe S., Lee S. H., Noda K., Nakamori S., Gu J., Ikeda Y., Kuroki Y., Sengoku K., Ishikawa M., Taniguchi N. (2009). Core fucosylation
of E-cadherin enhances cell-cell
adhesion in human colon carcinoma WiDr cells. Cancer Sci..

[7] Kamio K., Yoshida T., Gao C., Ishii T., Ota F., Motegi T., Kobayashi S., Fujinawa R., Ohtsubo K., Kitazume S., Angata T., Azuma A., Gemma A., Nishimura M., Betsuyaku T., Kida K., Taniguchi N. (2012). α1,6-Fucosyltransferase
(Fut8) is implicated in vulnerability to elastase-induced emphysema
in mice and a possible non-invasive predictive marker for disease
progression and exacerbations in chronic obstructive pulmonary disease
(COPD). Biochem. Biophys. Res. Commun..

[8] Fukuda T., Hashimoto H., Okayasu N., Kameyama A., Onogi H., Nakagawasai O., Nakazawa T., Kurosawa T., Hao Y., Isaji T., Tadano T., Narimatsu H., Taniguchi N., Gu J. (2011). α1,6-Fucosyltransferase-deficient
Mice Exhibit Multiple Behavioral Abnormalities Associated with a Schizophrenia-like
Phenotype: importance of the balance between the dopamine and serotonin
systems. J. Biol. Chem..

[9] Lv Y., Zhang Z., Wang M., Wang Y., Chen M., Jia J., Guo Y., Wang K., Li Z., Wang W., Li H. (2024). Discovery of novel FUT8 inhibitors with promising affinity and in
vivo efficacy for colorectal cancer therapy. Bioorg. Chem..

[10] Ihara H., Ikeda Y., Toma S., Wang X., Suzuki T., Gu J., Miyoshi E., Tsukihara T., Honke K., Matsumoto A., Nakagawa A., Taniguchi N. (2007). Crystal structure of mammalian α1,6-fucosyltransferase,
FUT8. Glycobiology.

[11] Ihara H., Okada T., Taniguchi N., Ikeda Y. (2020). Involvement of the
α-helical and Src homology 3 domains in the molecular assembly
and enzymatic activity of human α1,6-fucosyltransferase, FUT8. Biochim. Biophys. Acta Gen. Subj..

[12] Garcia-Garcia A., Ceballos-Laita L., Serna S., Artschwager R., Reichardt N. C., Corzana F., Hurtado-Guerrero R. (2020). Structural
basis for substrate specificity
and catalysis of α1,6-fucosyltransferase. Nat. Commun..

[13] Ramirez A. S., Boilevin J., Mehdipour A. R., Hummer G., Darbre T., Reymond J. L., Locher K. P., Hummer G. (2018). Structural basis of the molecular ruler mechanism of
a bacterial glycosyltransferase. Nat. Commun..

[14] Takahashi T., Ikeda Y., Tateishi A., Yamaguchi Y., Ishikawa M., Taniguchi N. (2000). A sequence
motif involved in the
donor substrate binding by alpha1,6-fucosyltransferase: the role of
the conserved arginine residues. Glycobiology.

[15] Garcia-Garcia A., Serna S., Yang Z., Delso I., Taleb V., Hicks T., Artschwager R., Vakhrushev S. Y., Clausen H., Angulo J., Corzana F., Reichardt N. C., Hurtado-Guerrero R. (2021). FUT8-Directed Core Fucosylation of
N-glycans Is Regulated
by the Glycan Structure and Protein. Environment
ACS Catal..

[16] Koetzler M. P., Blank S., Bantleon F. I., Spillner E., Meyer B. (2012). Donor substrate
binding and enzymatic mechanism of human core α1,6-fucosyltransferase
(FUT8). Biochim. Biophys. Acta, Gen. Subj..

[17] Lira-Navarrete E., Valero-Gonzalez J., Villanueva R., Martinez-Julvez M., Tejero T., Merino P., Panjikar S., Hurtado-Guerrero R. (2011). Structural
insights into the mechanism of Protein O-fucosylation. PLoS One.

[18] Sanz-Martínez I., García-García A., Tejero T., Hurtado-Guerrero R., Merino P. (2022). The Essential Role
of Water Molecules in the Reaction
Mechanism of Protein O-Fucosyltransferase 2. Angew. Chem., Int. Ed..

[19] Piniello B., Lira-Navarrete E., Takeuchi H., Takeuchi M., Haltiwanger R. S., Hurtado-Guerrero R., Rovira C. (2021). Asparagine Tautomerization in Glycosyltransferase
Catalysis. The Molecular Mechanism of Protein O-Fucosyltransferase
1. ACS Catal..

[20] Tvaroška I. (2015). Atomistic
insight into the catalytic mechanism of glycosyltransferases by combined
quantum mechanics/molecular mechanics (QM/MM) methods. Carbohydr. Res..

[21] Adero P. O., Amarasekara H., Wen P., Bohé L., Crich D. (2018). The Experimental Evidence in Support
of Glycosylation Mechanisms
at the S­(N)­1-S­(N)­2 Interface. Chem. Rev..

[22] Johnson E.
R., Keinan S., Mori-Sanchez P., Contreras-Garcia J., Cohen A. J., Yang W. (2010). Revealing
Noncovalent
Interactions. J. Am. Chem. Soc..

[23] b Grin, Y. ; Savin, A. ; Silvi, B. The Chemical Bond: Fundamental Aspects of Chemical Bonding; Frenking, G. , Shaik, S. , Eds.; Wiley VCH: Weinheim, 2014; p 345–382.

[24] Ortega A., Manzano R., Uria U., Carrillo L., Reyes E., Tejero T., Merino P., Vicario J. L. (2018). Catalytic Enantioselective Cloke–Wilson Rearrangement. Angew. Chem., Int. Ed..

[25] Fang D., Chaudret R., Piquemal J. P., Cisneros G. A. (2013). Toward
a Deeper Understanding of Enzyme Reactions Using
the Coupled ELF/NCI Analysis: Application to DNA Repair Enzymes. J. Chem. Theory Comput..

[26] Kraka E., Cremer D. (2010). Computational Analysis
of the Mechanism of Chemical Reactions in Terms of Reaction Phases:
Hidden Intermediates and Hidden Transition States. Acc. Chem. Res..

[27] Garay G., Hurtado J., Pedrón M., García L., Reyes E., Sánchez-Díez E., Tejero T., Carrillo L., Merino P., Vicario J. L. (2023). Organocatalytic
Enantioselective Vinylcyclopropane-Cyclopentene (VCP-CP) Rearrangement. Angew. Chem., Int. Ed..

[28] Fu Y., Bernasconi L., Liu P. (2021). Ab Initio Molecular Dynamics Simulations
of the SN1/SN2Mechanistic Continuum in Glycosylation Reactions. J. Am. Chem. Soc..

[29] Bohé L., Crich D. (2016). Glycosyl Cations out on Parole. Nat. Chem..

[30] Kozmon S., Tvaroska I. (2006). Catalytic mechanism of glycosyltransferases: hybrid
quantum mechanical/molecular mechanical study of the inverting N-acetylglucosaminyltransferase
I. J. Am. Chem. Soc..

[31] Lassila J. K., Zalatan J. G., Herschlag D. (2011). Biological
phosphoryl-transfer reactions: understanding mechanism and catalysis. Annu. Rev. Biochem..

[32] Case, D. A. ; Ben-Shalom, I. Y. ; Brozell, S. R. ; Cerutti, D. S. ; III, T. E. C. ; Cruzeiro, V. W. D. ; Darden, T. A. ; Duke, R. E. ; Ghoreishi, D. ; Gilson, M. K. ; Gohlke, H. ; Goetz, A. W. ; Greene, D. ; Harris, R. ; Homeyer, N. ; Huang, Y. ; Izadi, S. ; Kovalenko, A. ; Kurtzman, T. ; Lee, T. S. ; LeGrand, S. ; Li, P. ; Lin, C. ; Liu, J. ; Luchko, T. ; Luo, R. ; Mermelstein, D. J. ; Merz, K. M. ; Miao, Y. ; Monard, G. ; Nguyen, C. ; Nguyen, H. ; Omelyan, I. ; Onufriev, A. ; Pan, F. ; Qi, R. ; Roe, D. R. ; Roitberg, A. ; Sagui, C. ; Schott-Verdugo, S. ; Shen, J. ; Simmerling, C. L. ; Smith, J. ; Salomon-Ferrer, R. ; Swails, J. ; Walker, R. C. ; Wang, J. ; Wei, H. ; Wolf, R. M. ; Wu, X. ; Xiao, L. ; York, D. M. ; Kollman, P. A. AMBER 2023; University of California: San Francisco, 2023.

[33] Lu Y., Sen K., Yong C., Gunn D. S. D., Purton J. A., Guan J., Desmoutier A., Abdul Nasir J., Zhang X., Zhu L., Hou Q., Jackson-Masters J., Watts S., Hanson R., Thomas H. N., Jayawardena O., Logsdail A. J., Woodley S. M., Senn H. M., Sherwood P., Catlow C. R. A., Sokol A. A., Keal T. W. (2023). Multiscale
QM/MM modelling of catalytic systems with
ChemShell. Phys. Chem. Chem. Phys..

[34] Frisch, M. J. ; Trucks, G. W. ; Schlegel, H. B. ; Scuseria, G. E. ; Robb, M. A. ; Cheeseman, J. R. ; Scalmani, G. ; Barone, V. ; Petersson, G. A. ; Nakatsuji, H. ; Li, X. ; Caricato, M. ; Marenich, A. V. ; Bloino, J. ; Janesko, B. G. ; Gomperts, R. ; Mennucci, B. ; Hratchian, H. P. ; Ortiz, J. V. ; Izmaylov, A. F. ; Sonnenberg, J. L. ; Williams; Ding, F. ; Lipparini, F. ; Egidi, F. ; Goings, J. ; Peng, B. ; Petrone, A. ; Henderson, T. ; Ranasinghe, D. ; Zakrzewski, V. G. ; Gao, J. ; Rega, N. ; Zheng, G. ; Liang, W. ; Hada, M. ; Ehara, M. ; Toyota, K. ; Fukuda, R. ; Hasegawa, J. ; Ishida, M. ; Nakajima, T. ; Honda, Y. ; Kitao, O. ; Nakai, H. ; Vreven, T. ; Throssell, K. ; Montgomery, Jr, J. A. ; Peralta, J. E. ; Ogliaro, F. ; Bearpark, M. J. ; Heyd, J. J. ; Brothers, E. N. ; Kudin, K. N. ; Staroverov, V. N. ; Keith, T. A. ; Kobayashi, R. ; Normand, J. ; Raghavachari, K. ; Rendell, A. P. ; Burant, J. C. ; Iyengar, S. S. ; Tomasi, J. ; Cossi, M. ; Millam, J. M. ; Klene, M. ; Adamo, C. ; Cammi, R. ; Ochterski, J. W. ; Martin, R. L. ; Morokuma, K. ; Farkas, O. ; Foresman, J. B. ; Fox, D. J. Gaussian, Inc., GaussView 5.0. Wallingford, CT, 2009.

[35] Kühne T. D., Iannuzzi M., Del Ben M., Rybkin V. V., Seewald P., Stein F., Laino T., Khaliullin R. Z., Schütt O., Schiffmann F., Golze D., Wilhelm J., Chulkov S., Bani-Hashemian M. H., Weber V., Borštnik U., Taillefumier M., Jakobovits A. S., Lazzaro A., Pabst H., Müller T., Schade R., Guidon M., Andermatt S., Holmberg N., Schenter G. K., Hehn A., Bussy A., Belleflamme F., Tabacchi G., Glöß A., Lass M., Bethune I., Mundy C. J., Plessl C., Watkins M., VandeVondele J., Krack M., Hutter J. (2020). CP2K: An electronic
structure and molecular dynamics software package - Quickstep: Efficient
and accurate electronic structure calculations. J. Chem. Phys..

[36] Tribello G. A., Bonomi M., Branduardi D., Camilloni C., Bussi G. (2014). PLUMED2: New feathers for an old
bird. Comput.
Phys. Commun..

