# Supplementary material for: FUT8 Catalysis Involves GDP-Fucose–Induced Loop Activation Promoting a Reaction at the SN1‑SN2 Frontier
Source: ACS Catal. 2025 Dec 19;16(4):3534–46. doi: 10.1021/acscatal.5c07826 (PMC12930516; doi:10.1021/acscatal.5c07826)
Supplement: Supplementary file 1 [file cs5c07826_si_001.pdf]

FUT8 Catalysis Involves GDP-fucose–Induced Loop Activation Promoting a Reaction at the S_N1-S_N2 Frontier

Ignacio Sanz-Martínez,[a,b] *Tomás Tejero,*[b,c] *Ramón Hurtado-Guerrero**[a,d,e] *and Pedro Merino**[a,b].

[a] Institute of Biocomputation and Physics of Complex Systems (BIFI), University of Zaragoza 50018, Zaragoza, Spain

[b] Department of Organic Chemistry. Faculty of Sciences, University of Zaragoza, 50009, Zaragoza, Spain.

[c] Institute of Chemical Synthesis and Homogeneous Catalysis (ISQCH), University of Zaragoza-CSIC, 50009 Zaragoza, Spain

[d] Copenhagen Center for Glycomics, Department of Cellular and Molecular Medicine, University of Copenhagen, Blegdamsvej 3B, Copenhagen 2200, Denmark

[e] Fundacion ARAID, Zaragoza, Spain

E-mail: pmerino@unizar.es
rhurtado@bifi.es

Supporting Information

1 Table of Contents

1 Table of Contents 1
2 Molecular Dynamics Simulations 2
2.1 Model building 4
2.2 Stabilization of GDP-Fucose 5
2.3 Loop dynamics 7
3 QM/MM calculations 8
3.1 Charge deletion analysis 10
3.2 Energies 12
4 QM/MM//MD metadynamics simulations 14
4.1 General Data 14
4.2 Free Energy Landscapes (FEL) of Carbohydrate Units 15
4.2.1 Fucose Free Energy Landscape (FEL) 15
4.2.2 Innermost GlcNAc (G0A) Free Energy Landscape (FEL) 16
4.2.3 Fucose Transfer Free Energy Surface (FES) 17

4.3 Conformational behavior of involved sugar units 20
4.4 The catalytic cycle concerning the fucose transfer 21
5 Topological calculations (ELF & NCI) 23
5.1 NCI (Non-Covalent Interactions) 23
5.2 ELF (Electron Localization Function) 23
5.2.1 ELF analysis for the minimum energy path 23
5.2.2 ELF analyses for MD trajectories 31
6 Additional supporting material 31
6.1 PDB Files 32
6.2 Video Files 32
Descriptors-ELF.mp4 32
Reaction.mp4 32
7 References 33

2 Molecular Dynamics Simulations

Molecular Dynamic (MD) simulations were carried out with AMBER23 package[1] implemented with FF14SB[2] and GAFF[3] force fields. The parameters and charges GDP-fucose were generated with the antechamber module of AMBER using GAFF force field and AM1-BCC method for charges, and those for the acceptor G0 were generated with GLYCAM06.[4] The guess structure for the simulation was generated with the XLeap module of AMBER23 and was then immersed in an orthorhombic water box with a 18 Å buffer of TIP3P water molecules.[5] The system was neutralized by adding explicit counterions (Na^+). A standardized protocol was then performed: (i) 5000 steps of energy minimization of solvent molecules was performed, consisting of 2500 steps using the steepest descent algorithm and 2500 steps with the conjugate gradient method, and applying restraints to the protein and ligands. (ii) Unrestrained additional 5000 minimization steps were executed under the same conditions. (iii) The systems were then gently heated by incrementing the temperature from 0 to 300 K under a constant pressure of 1 atm and periodic boundary conditions. Harmonic restraints of 30 kcal/mol were applied to the solute, and the Langevin temperature-coupling scheme[6] was used to control and equalize the temperature. The timestep was kept at 1 fs during the heating stages, allowing potential inhomogeneities to self-adjust. Long-range electrostatic effect was modelled using the particle-mesh-Ewald method. [7] An 8 Å cut-off was applied to Lennard-Jones interactions. (iv) The systems were equilibrated for 2 ns with a 2 fs time step at a constant volumen and temperature 300 K. To ensure system stability this step was repeated. (v) Production trajectories were then run for additional 1000 ns under periodic boundary conditions at a constant volume and temperature of 300 K using the Langevin thermostat, with a collision frequency of 2.0 ps^{-1}. Chemical

bonds involving hydrogen atoms were constrained using the SHAKE algorithm, [8] and the equations of motion were integrated with a 2 fs timestep. To ensure adequate conformational sampling, five independent replicas were performed for each model, each initiated with a randomly generated velocity distribution. [9]

Typical input files for these calculations are:

(i) First minimization

```
Minimization-1
 &cntrl
 imin   = 1,    ntb   = 1,
 maxcyc = 10000, ncyc  = 5000,
 igb    = 0,    ntr   = 1,      cut  = .0,
/
Substrate fixed
500.0
RES 1 478
END
END
```

(ii) Second minimization

```
Minimization-2
 &cntrl
 imin   = 1,     ntb   = 1,
 maxcyc = 10000, ncyc  = 5000,
 igb    = 0,      cut  = .0,
/
```

(iii) Heating

```
100 ps MD res on solute
 &cntrl
 imin   = 0,     irest  = 0,
 ntx    = 1,     ntb    = 1,     ntc   = 2,
 ntf    = 2,     ntr    = 1,     cut   = 8.0,
 vlimit = 10,    gamma_ln = 10,
 tempi  = 0.0,   temp0  = 300.0,
 ntt    = 3,     ioutfm = 1,     iwrap = 1,
 nstlim = 150000, dt     = 0.001,
 ntpr   = 100,   ntwx   = 100,   ntwr  = 1000,
/
Keep solute fixed with weak restraints
10.0
RES 1 478
END
END
```

(iv) Equilibration

```
100 ps MD res on solute
 &cntrl
 imin   = 0,    irest  = 1,
 ntx    = 7,    ntb    = 2,    ntc   = 2,
 ntf    = 2,    ntr    = 0,    cut   = 8.0,
 tempi  = 300.0, temp0  = 300.0, pres0 = 1.0,
 ntt    = 1,    ntp    = 1,    taup  = 2.0,
 nstlim = 50000, dt     = 0.002,
 ntpr   = 10,   ntwx   = 100,  ntwr  = 10,
/
```

(v) Production

```
200 ns in MD
 &cntrl
 imin   = 0,    irest  = 1,
 ntx    = 7,    ig     = 14292,
 ntb    = 2,    cut    = 8.0,
 ntr    = 0,    ntc    = 2,   ntf  = 2,
 tempi  = 300.0, temp0=300.0,  ntt  = 3,
 gamma_ln = 2.0,
 pres0  = 1.0,   ntp = 1,     taup = 2.0,
 nstlim = 100000000,   dt = 0.002,
 ntpr   = 2000,  ntwx = 5000,  ntwr = 10000,
/
```

2.1 Model building

The model of the Michaelis complex (MC) composed from the protein in its active conformation, hexasaccharide G0, and GDP-fucose was constructed starting from the crystal structure of the ternary complex formed by FUT8 in its active form, the hexasaccharide G0 and GDP (PDB ID: 6TK).[10] Notably, the peptide portion of the fucose-accepting glycopeptide does not play a significant role in the catalytic process. The most complete chain of the dimer (chain A) was selected, and its structure was completed using the Modeller tool as implemented in UCSF chimera. [11] Subsequently, a GDP-fucose molecule was positioned by aligning its GDP moiety with the nucleoside present in the original crystal structure, providing the guest structure for MD simulations which were carried out as described above.

For structures in which the enzyme is in its inactive form, with both loops in an open conformation, the crystal structure of FUT8 in its *apo* form (PDB ID: 2DE0) [12] was used. Its structure was completed using the Modeller tool as implemented in UCSF chimera.[11] Subsequently, the corresponding ligand was introduced into the active site by the alignment with the previously generated ternary complex using the align tool in PyMOL 3.0.[13] All the remaining models used in this study were generated through alignment and modification of these two initial models. For the analysis of mutants, the structures

were derived from the original models by substituting specific residues using the mutagenesis tool in PyMOL. In all cases, protonation states were assigned based on predictions obtained from PROPKA3.[14] Coordinate root-mean-squared deviation (RMSD) analyses were made for all the MD simulations using RMS command as implemented in CPPTRAJ[15] (AMBER) taking as reference the first frame. Other analyses regarding distnaces between atoms or center of mass were made with CPPTRAJ[15] (AMBER). All the molecular representations were made with PyMOL 3.0.[13] Graphic linear representations were made using GNUplot software.[16]

PDB files containing starting and final structured coordinates are given for simulations of *apo* form (inactive) -trajectory-01a,b-, close conformation CC (active) -trajectory-02a,b-, close conformation in complex with GDP-Fucose -trajectory-03a,b- and the Michaelis complex MC -trajectory-04a,b-.

Along this supporting information we will use the following nomenclature for the atoms involved in the studied reaction:

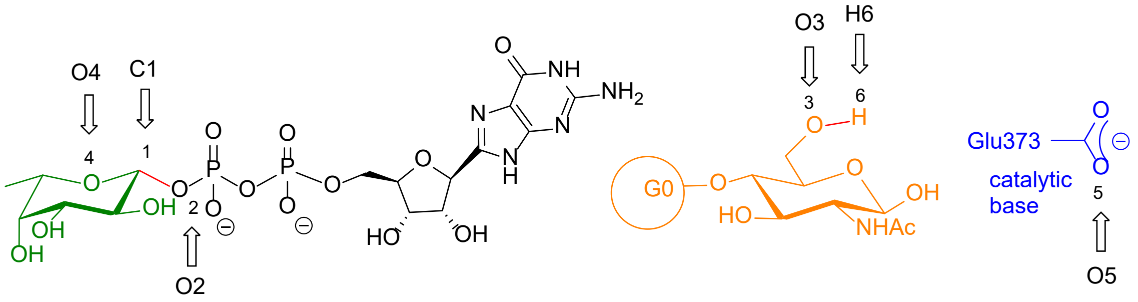

2.2 Stabilization of GDP-Fucose

The interactions observed between GDP-Fucose and FUT8 were tracked using the HBOND and LIFETIME commands as implemented in CPPTRAJ[15]. The HBOND command calculates h-bonds using geometric criteria and can provide H-bonds average. Only solute-solute h-bonds were considered. The criteria used for tracking H-bonds were those implemented in AMBER23. The LIFETIME command performs lifetime analysis along the trajectory for specified data sets, in our case the found H-bonds. The analysis was performed with five different replicas, selecting interactions with a persistence percentage of 60% or higher. In cases where atoms can exchange positions (oxygen atoms of the carboxyl group in the side chains of Asp and Glu (OD1 & OD2); HZ atoms of Lys or NH groups in Arg), both atoms were considered equivalent. Standard deviations between the different replicas were calculated. We found persistent interactions (more than 60% of the trajectory) with Ser469, Asp453, Arg365, Lys369, and Tyr220 (Figure S1, a). Interactions between loops are maintained in the simulations (Figure S1, b). In the case of mutants D368A and R441A, the interactions with Lys369 and Arg365 are maintained (Figure S1, c).

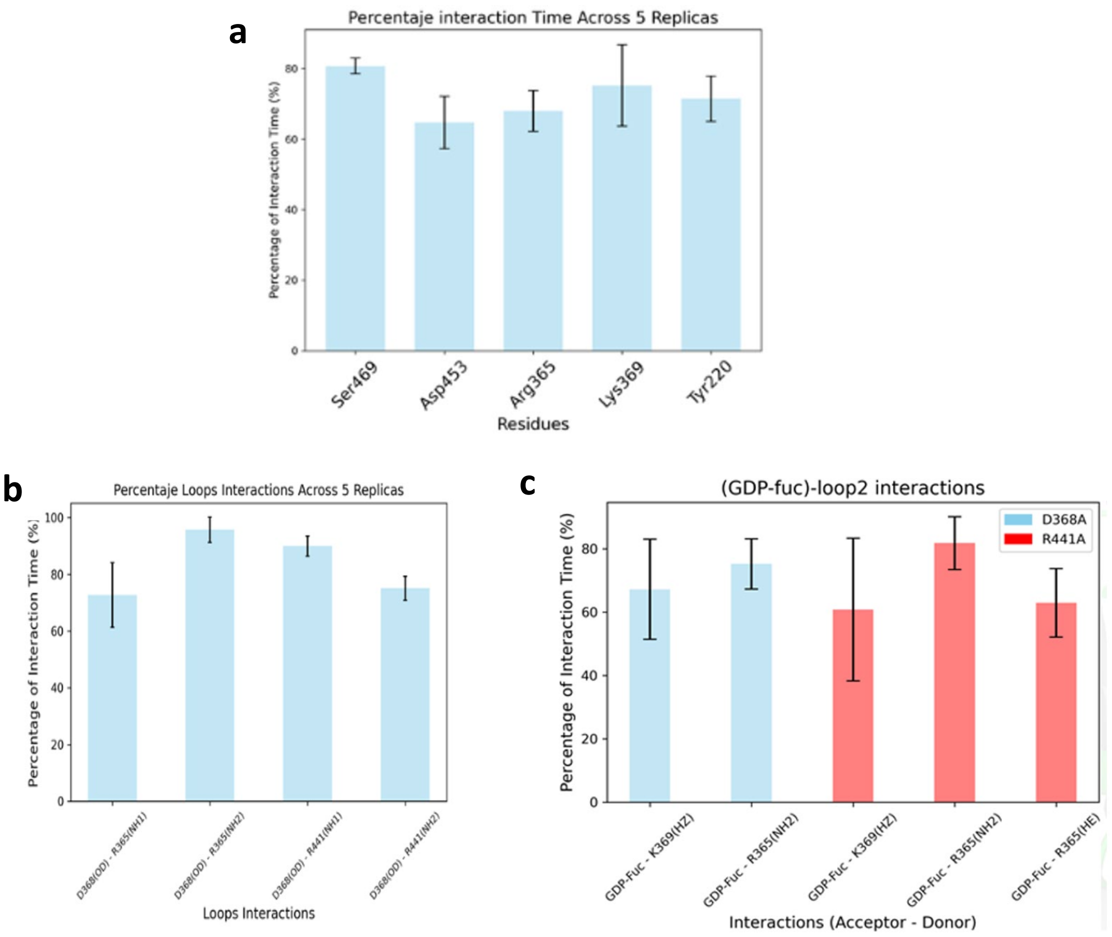

Figure S1. (a) Bar plot showing the percentage of hydrogen bond occupancy between GDP-Fuc and key residues across simulations (error bars = standard deviations). Bar plot showing the percentage of hydrogen bond occupancy for key residues across simulations (error bars = standard deviations). (c) Bar plot representing the hydrogen bond occupancy of GDP-Fuc with key loop 2 residues in the D368A and R441A mutants. (error bars = standard deviations)

The interaction between Asp368 and GDP-fucose was studied in detail. In particular, we studied the evolution of the distance between Asp368 and GDP-fucose, and it was combined for five replicas to ensure reproducibility. The minimum distance between the oxygen atoms (OD) (the carboxyl group rotates easily) of residue Asp368 and the hydroxyl group (H4O) of fucose was monitored at 1-nanosecond (ns) intervals during the five simulation replicas performed. For each time point (every ns), the corresponding distance values across the five replicas were calculated, and the standard deviation of these measurements was subsequently obtained. In this way, a graph was generated representing the temporal evolution of the average distance between Asp368 and the OH4 group of fucose, along with its corresponding standard deviation (grey strip).

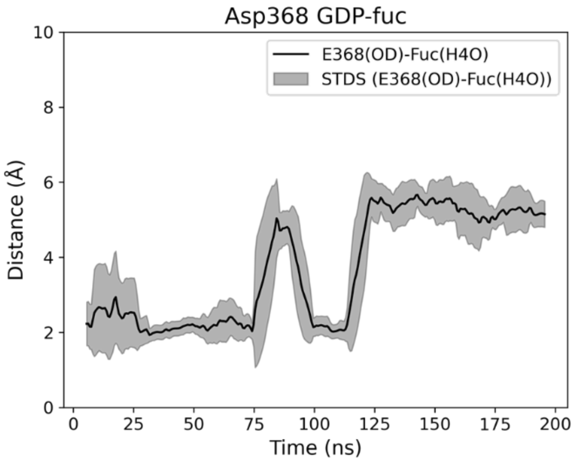

Figure S2. Evolution of Asp368-H4O distance along 5 Replicas. The grey band represents the standard deviation

2.3 **Loop dynamics**

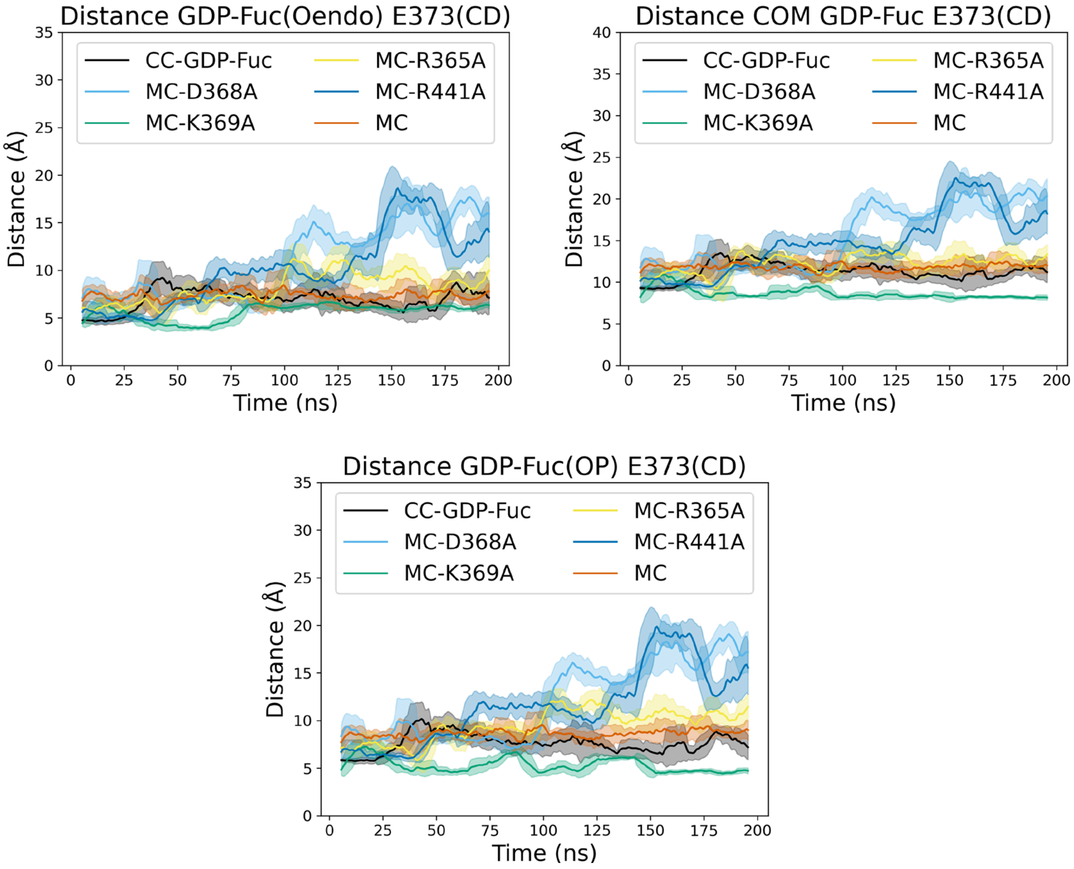

Figure S3. Distances between the carboxylic carbon (CD) of Glu373 and (a) the endo oxygen of the fucose in GDP-fucose, (b) center of mass of GDP-fucose, and (c) the Oxygen atom of the pyrophosphate linked to the anomeric carbon in the different complexes studied

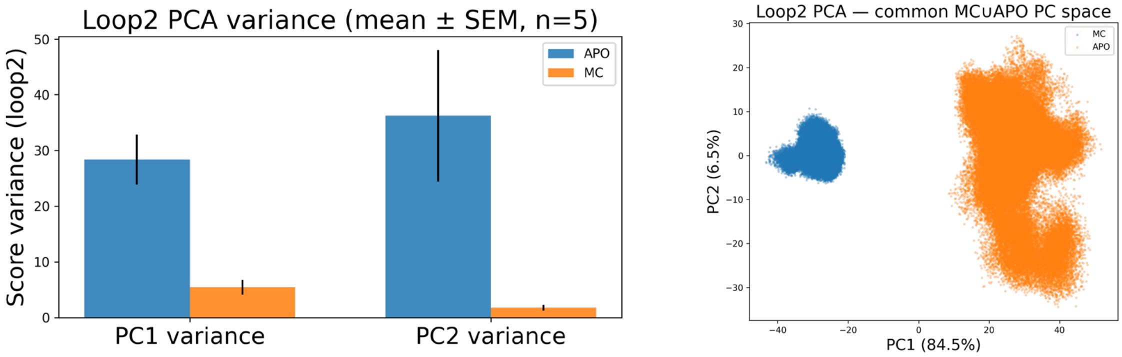

Figure S4. Principal component analysis (PCA) analysis of the *apo* form and the Michaelis complex

3 QM/MM calculations

Restrained MD simulations of involved distances d_1-d_4 starting from previous normal MD simulations of the Michaelis complex were performed to generate molecular configurations depicting the nucleophilic attack of O3(G0) to the anomeric carbon of the fucose, departure of the phosphate group and H-transfer to Glu373. All the generated structures were equilibrated by at least 500 ns. The equilibrated systems were used as guest structures for QM/MM calculations. after removing the solvent, leaving only a sphere of waters of 22 Å, centered on the anomeric carbon of the fucose unit. We selected 115 atoms described accurately with quantum chemistry (Figure S5). A non-periodic electronic embedding scheme was used as implemented in the ChemShell software.[17] Noteworthy, no water molecules were found in the vicinity of the active site that could directly participate in the reaction as previously reported for POFUT2.[18] Any attempt to include between three and six water molecules in the proper positions similar to we had observed in POFUT2, resulted in unstable configurations and/or potential energy surfaces (PES) lacking a clear transition state. Therefore, models obtained from MD simulations that did not include any water molecules. The system was partitioned into quantum and classical regions. The QM region (Figure S5, b) comprised the side chains of Glu373 (catalytic base), Arg365, and Lys369, with the latter two stabilizing the diphosphate through a salt bridge, the L-fucose linked by the pyrophosphate unit, and the acceptor Glc-NAc, along with the C5 and O5 atoms and their substituents from the adjacent Glc-NAc. All cuts between QM and MM regions were applied in aliphatic C-C bonds. For hexasaccharide G0, the QM/MM partitioning was defined between the C5-C4 and C1-C2 bonds of the second Glc-NAc, accepting potential overpolarization errors due to the lack of options for partitioning aliphatic chains.[19] In total, the QM region comprised 115 atoms with a net charge of -1 a.u. directly involved in the reaction were used as starting points.

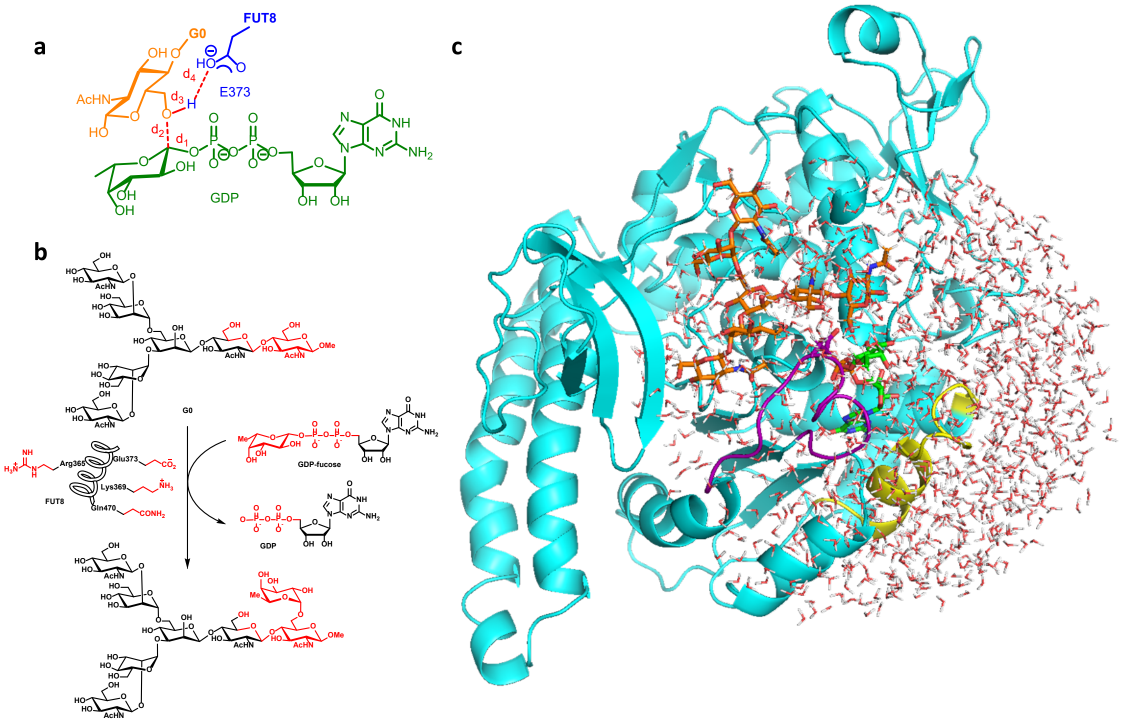

Figure S5. QM/MM calculations. (a) Involved breaking/forming bonds. (b) Red atoms indicate QM atoms and black atoms the active region. (c) Full view of the optimized reagent showing the sphere of waters. Hexasaccahride G0 is colored in orange, GDP-fucose in green, loop1 in yellow and loop2 (containing the catalytic base, showed in sticks) in purple.

The QM region was treated with BP86 hamiltonian[20] combined with SVP basis set[21] using correction for the dispersion, a level of theory that was demonstrated to provide good geometries and accurate energy values within reasonable computational times.[22] The rest of the atoms were allowed to move during the optimization process (active region) and they (MM part) were described using the Amber ff14SB9[2] and gaff212[3] force fields, along with the TIP3P water model for the solvent. An electronic embedding scheme[22] was used for the QM/MM treatment. All QM/MM calculations were performed with the modular program package ChemShell,[23] using Gaussian09[24] to obtain the quantum mechanical (QM) energies and gradients at the SCC-DFTB (Self-Consistent Charge Density Functional Tight-Binding) level.[25] No cutoffs were introduced for the non-bonding MM and QM/MM interactions. In previous works, it has been shown that the BP86 functional was a good choice for an initial approach to glycosylation reactions with Leloir's nucleotides and that the QM(BP86/SVP)/AMBER level of calculation provided good geometries and energies at reasonable computational time. The energies and gradients for the MM region were evaluated by DL_POLY,[26] which was accessed through the ChemShell package using the AMBER23 parameters. The QM/MM optimizations were performed using the limited-memory Broyden–Fletcher–Goldfarb–Shanno (LBFGS)[27] algorithm combined with the Hybrid Delocalized Internal Coordinate Scheme.[28] The reaction paths were scanned by performing harmonically restrained optimizations along a suitable reaction coordinate (see below) in steps of 0.2 Å. For each

potential energy profile, the highest potential energy structure was taken as an approximation to the corresponding transition state structure.

We studied the potential energy surface (PES) by varying the different distances involved in the reaction (Figure S6a). Thus, the reaction mechanism was monitored using reaction coordinate constructed from the distance between the anomeric carbon C1 and glycosidic oxygen O1 of the pyrophosphate unit of GDP-fucose, representing the release of the leaving group. Scans using other distances showed in Figure S1 (d2, d3 or d4) or combination of them, did not produce neither reasonable reaction coordinates nor transition structure. When the distance from the oxygen atom of the acceptor G0 to the anomeric carbon of the fucose was monitored a smooth PES was obtained (Figure S6b), from which we located and optimized the starting and the final points of the reaction, **RE$_{QM}$** and **PR$_{QM}$**, respectively, as well as the corresponding transition structure **TS$_{QM}$** (Figure S6c) Coordinates of optimized **RE$_{QM}$**, **TS$_{QM}$** and **PR$_{QM}$** are provided as pdb files. The transition state was identified using the dimer approach, selecting initial structures from different points along the generated potential energy surface. the analysis of the potential energy surface (PES), through the monitoring of interatomic distances along the reaction, confirmed a late transition state for an asynchronous reaction in which the phosphate group departs prior to the entry of the hydroxyl group, while H transfer occurs concomitantly with the formation of the glycosidic bond (Figure S6d).

3.1 Charge deletion analysis

The estimation of the contribution of individual residues to the stabilization/destabilization of the transition structure was carried out through a charge deletion analysis.[29] This analysis is made for residues surrounding the QM area so, they should not form a part of the QM region, but of the active region. For the charge deletion analysis, atomic charges for each residue were extracted using the following ChemShell script command:

types set fp [open ${aminoacid}.txt a] puts $fp "${atom_charges}" close $fp

The resulting file provided the atomic charges associated with each residue. For the selected residues, the atomic charges were manually set to zero to simulate the deletion of their electrostatic contribution. The modified charge file was subsequently used as input for single-point energy calculations to assess the energetic impact of each residue. All charge deletion and energy evaluation procedures were performed using the ChemShell software package.[17] We considered as relevant residues those around 3.5 Å from the active site and those of the loops 1 and 2. A negative value indicates destabilization and a positive value indicates stabilization (Figure S6e). Values above 1-2 kcal/mol (absolute values) are considered relevant.

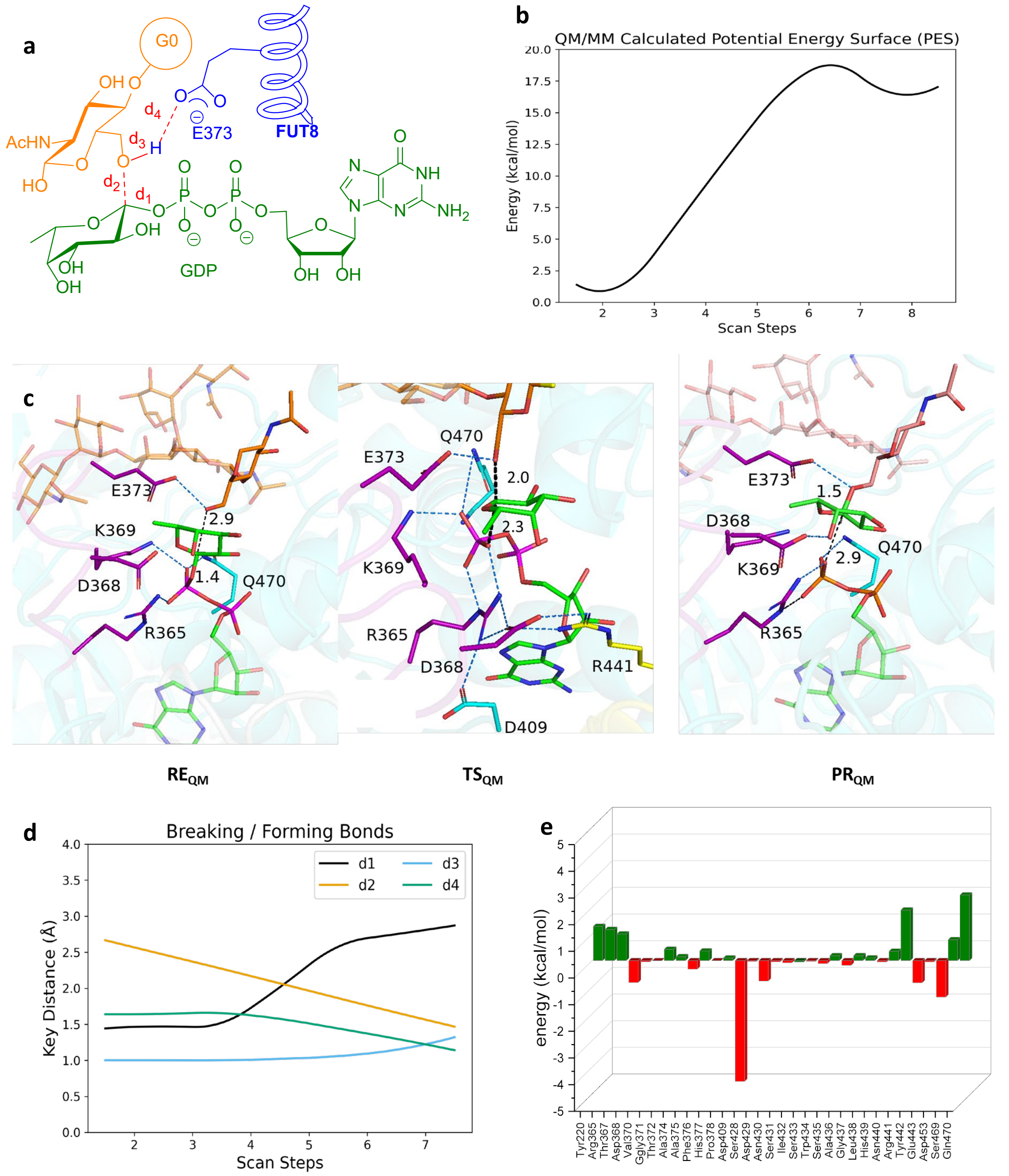

Figure S6. QM/MM calculations. (a) Key distances involved in the glycosylation reaction. (b) Potential Energy Surface (PES) for d1. (c) Stationary points(reactant **RE$_{QM}$**, transition structure **TS$_{QM}$** and product **PR$_{QM}$**. Geometries optimized at bp86/def2svp level of theory. Hydrogens have been omitted for clarity. Interactions between residues, GlcNAc and fucose showing the H-bond network in the transition structure. Protein is colored in cyan; residues from loop1 and loop2 are colored in yellow and purple, respectively. (d) Breaking and forming bonds during the reaction (QM/MM). (e) Charge deletion analysis of **TS$_{QM}$** at BP86/def2tzvp level of theory.

A small stabilization of 2.5 kcal/mol can be assigned to Gln470 a residue contributing to the correct positioning of the substrate. The destabilization observed for Asp409 (–4.5 kcal/mol) and in a minor extent Asp368 (-0.82 kcal/mol), is the same as that observed

with POFUT2[18] and is due to a hydrogen bond interaction with the guanidine group of Arg365 (Figure 4c), harming the hydrogen bond with the leaving pyrophosphate group. Presumably, this is a price that must be paid to keep Arg365 in the proper position to interact with the pyrophosphate, even if the electronic nature of the interaction is not optimal. In this respect, interactions between Arg441 and Asp368 compensates in some extent (1.9 kcal/mol) the influence of the later (Figure 4e). The inclusion of Arg441, Gln470, or Asp409 in the QM region resulted in negligible differences, confirming that the QM/MM partition used satisfactorily describes the system.

3.2 Energies

Energies of the three stationary points (Table S1) were obtained from the corresponding out files once convergence had been reached Figure S4.

Table S1. QM/MM energy values (kcal/mol) for the reaction pathway calculated at BP86/SVP levels of theory. (MM subsystem characterized with AMBER24).

Entry	Converged energy (hartree)	Relative energy (kcal/mol)
RE_{QM}	-3087.445198128767	
TS_{QM}	-3087.420080281665	15.8
PR_{QM}	-3087.427128972046	11.3

Single point calculations of RE_{QM} and TS_{QM} at several levels of theory were performed to evaluate the barriers obtained from the difference of those values. The results are collected in Table S2. The initial reaction barrier was calculated to be 15.9 kcal/mol; however, more accurate calculations made at 3ξ level (Table S2) showed energy barriers in the range 18.0-19.7 kcal/mol for GGA functionals with no HF exchange (BP86, PBE, BLYP and TPSS) (Table S2, entries 1-9). As we observed previously, hybrid-GGA methods like m062x furnished higher energies in agreement with other benchmark studies.[30] These values are in good agreement with those observed experimentally $k_{cat} \approx 10\ s^{-1}$ corresponding to ΔG‡ ≈ 16.5–17 kcal/mol and $k_{cat} \approx 0.25\ s^{-1}$ corresponding to ΔG‡ ≈ 19–20 kcal/mol.[17,31] However, the process under study is itself endergonic by 11.3 kcal/mol, due to the fact that the species immediately formed after the addition of the fucose unit has the catalytic base Glu373 in its protonated form, while the pyrophosphate carries three negative charges. The overall process must include an acid-base reorganization that restores the enzyme to its native state at physiological pH, that is, with Glu373 deprotonated and the pyrophosphate accepting the proton originally transferred from the alcohol during the nucleophilic attack. Under these conditions, a negative energy balance indicating a favored reaction, would be expected between $\mathbf{RE_{QM}}$ and the product in which the proton from G(0) has been transferred from the catalytic base to the pyrophosphate group. In this regard, no further calculations were pursued, and it was decided to study the system using QM/MM/MD (metadynamics simulations), which could provide a more realistic view of the overall process compared to QM/MM calculations which are based on a static (stationary) state

Table S2. QM/MM energy barriers (kcal/mol) for the SN2 reaction pathway calculated at various levels of theory. (MM subsystem characterized with AMBER24).

Entry	level of theory	Empirical coorection to dispersion	barrier (kcal/mol)
1	bp86-d3/6-311+G(d,p)	Yes	18.1
2	bp86-d3/cc-pvtz	Yes	18.0
3	bp86-d3/def2tzvp	Yes	18.5
4	tpsstpss-d3/6-311+G(d,p)	Yes	18.7
5	tpsstpss-d3/cc-pvtz	Yes	18.8
6	tpsstpss-d3/def2tzvp	Yes	19.1
7	blyp-d3/6-311+G(d,p)	Yes	19.4
8	blyp-d3/cc-pvtz	Yes	19.0
9	blyp-d3/def2tzvp	Yes	19.7
10	m062x-d3/6-311+G(d,p)	No	28.4
11	m062x-d3/cc-pvtz	No	27.7
12	m062x-d3/def2tzvp	No	27.7
13	PBE/6-311+G(d,p)	Yes	18.3
14	PBE/cc-pvtz	Yes	18.4
15	PBE/def2tzvp	Yes	18.6
16	M062x-d3/6-311+G(d,p)	No	28.4
17	m062x-d3/cc-pvtz	No	27.7
18	m062x-d3/def2tzvp	No	27.7

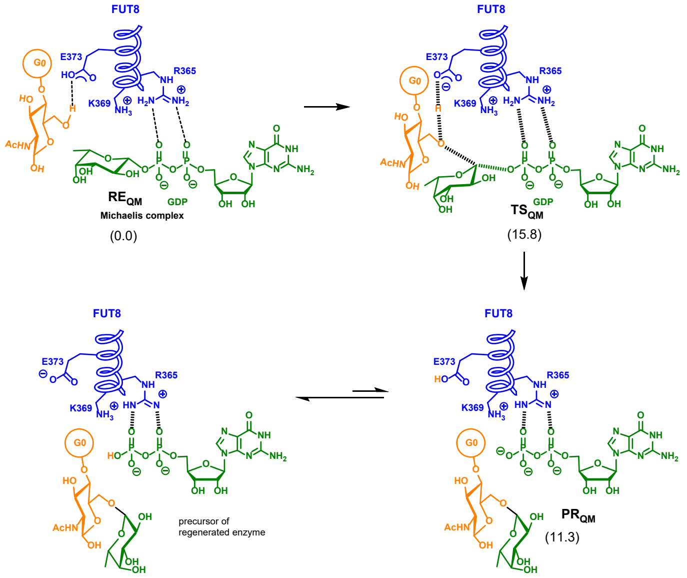

Figure S7. The full process should include a second proton-transfer from the catalytic base Glu373 to the pyrophosphate to complete the balance of the reaction and regenerate the enzyme.

4 QM/MM//MD metadynamics simulations

4.1 General Data

QM/MM metadynamics simulations were performed to analyze the conformational behavior of the two sugars involved in fucose transfer within the Michaelis complex (MC), to model the reaction mechanism, and to determine the free energy surface of the glycosylation reaction. For this purpose, the CP2K software[32] was used in combination with the metadynamics algorithm implemented in PLUMED 2.[33] The reactant obtained from stationary QM/MM calculations, was used as the starting point. The model included a total of 30341 water molecules and 6 Na^+ ions. The MM region was treated using the force fields (FF) employed in the MD simulations, while the QM region (Figure S8) was described at the DFT level using the PBE functional.[34] This choice aligns with previous studies on reaction mechanisms in glycosyltransferases[35] and carbohydrate conformations. [36]

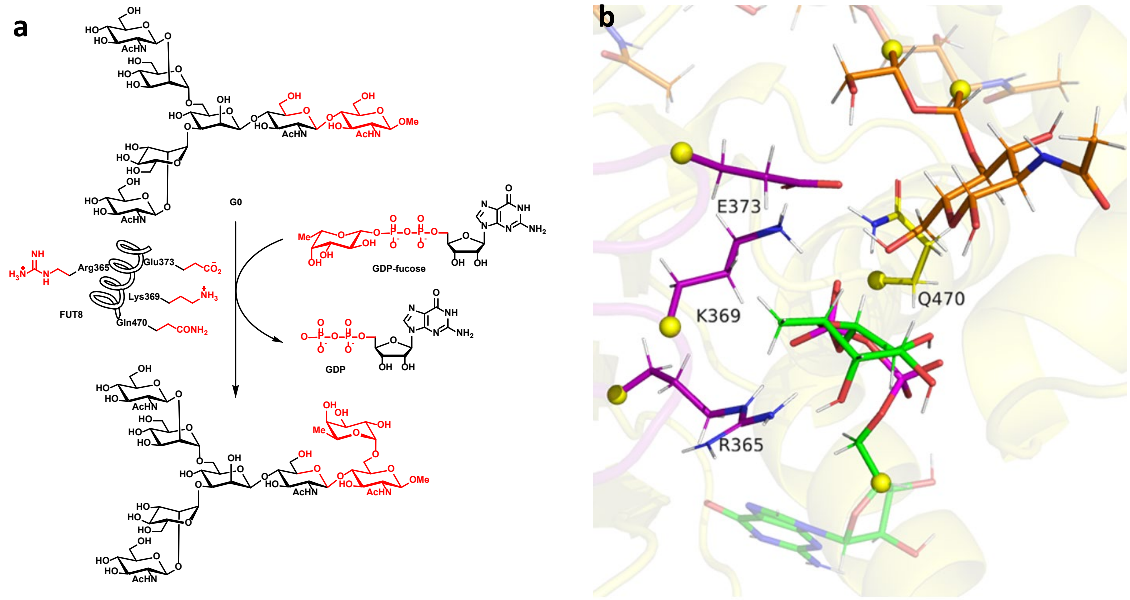

Figure S8. Metadynamics calculations. (a) Red atoms indicate QM atoms and black atoms the MM atoms. (b) QM region (solid colour) used in the QM/MM simulations for the Fucose Transfer Free energy Surface. Yellow spheres correspond to the capping hydrogen atoms. The protein is shown in yellow, with loop 1 in white, loop 2 in purple, GDP-Fuc in green, G0 in orange, G0-Fuc in pink, and GDP-H in gray.

The GPW (Gaussian and plane-waves) formalism was used with a mixed basis set approach. The Gaussian triple-ζ valence polarized (TZV2P) [37] basis set was employed to expand the wave function, while the electron density was converged using an auxiliary plane-wave basis set with a variable density cut-off, adjusted according to each studied model, along with GTH pseudopotentials.[38] All metadynamics simulations followed a standardized protocol: (i) a multi-step annealing process. (ii) a QM/MM simulation without an external potential at 300 K in an NVT ensemble for 5 ps, using a timestep of

0.5 fs, to equilibrate the system at the QM/MM level and (iii) the metadynamics simulations using as the starting point the final snapshot from the equilibration.

4.2 Free Energy Landscapes (FEL) of Carbohydrate Units

The conformational free energy landscape (FEL) of the innermost GlcNAc subunit from G0 and the fucose moiety from GDP-Fuc in the Michaelis complex was computed using collective variables derived from the Cremer-Pople puckering coordinates.[39] Specifically, Cartesian projection coordinates normalized by the Cremer-Pople puckering amplitude (Q) were used:

$$CV1 = \frac{qx}{Q}, \quad CV2 = \frac{qy}{Q}, \quad CV3 = \frac{qz}{Q}$$

To characterize the conformational behavior of both sugars, fucose and the innermost GlcNAc, the Cremer–Pople puckering coordinates (θ and ϕ) were calculated at each point along the minimum free energy path. The conformations were then represented on a Mercator projection, providing a detailed visualization of the structural evolution throughout the transfer process. In both cases, the reweighting of the Cremer-Pople puckering coordinates θ and ϕ was performed using PLUMED2. [33]

4.2.1 Fucose Free Energy Landscape (FEL)

The QM region included 33 atoms confined within an orthorhombic box with dimensions of 14.073 × 12.758 × 16.367 Å. These atoms comprised the fucose, the diphosphate, and the CH_2 group linking the ribose to the diphosphate. To complete the QM region, hydrogen atoms were added for capping. A plane-wave cutoff of 300 Ry was used, and Gaussian potentials were deposited every 30 fs with an initial height of 1.2 kcal/mol and widths of 0.035, 0.030, and 0.020 collective variable units (c.v.u.) for qx/Q, qy/Q, and qz/Q, respectively. To improve accuracy, the potential height was reduced to 0.6 kcal/mol after 300 ps (10000 added potentials) and extended for an additional 50 ps.

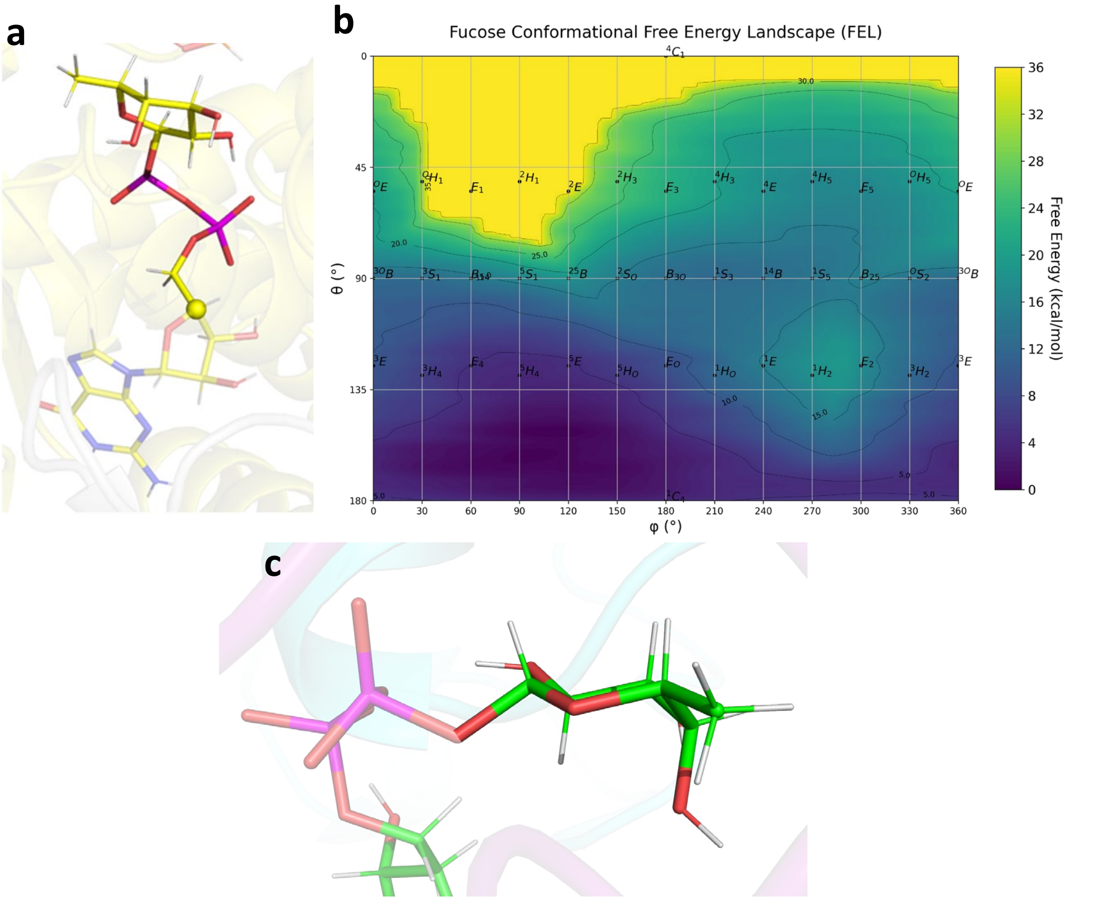

Figure S9. Metadynamics calculations. (a) QM region (solid colour) used in the QM/MM simulations for the fucose free energy landscape metadynamics. Yellow spheres correspond to the capping hydrogen atoms. The protein is shown in yellow, with loop 1 in white, loop 2 in purple, GDP-Fuc in green, G0 in orange, G0-Fuc in pink, and GDP-H in gray. (b) Free Energy Landscape (FEL) Analysis of Fucose within the MC. The lowest-energy regions indicate the preferred conformations. (c) Structural representation of fucose adopting the $^1C_4$ conformation (green) within the active site. The protein is shown in yellow, with loop 1 in white, loop 2 in purple, GDP-Fuc in green, and G0 in orange

4.2.2 Innermost GlcNAc (G0A) Free Energy Landscape (FEL)

For the innermost GlcNAc, the QM region included 49 atoms confined within an orthorhombic box with dimensions of 18.14 × 15.756 × 15.129 Å. This region contained the innermost N-acetylglucosamine, as well as the C1, C2, O5, and C5 atoms of the G0B ring along with their substituents. The QM region was capped with hydrogen atoms. A plane-wave cutoff of 290 Ry was used, and Gaussian potentials were deposited every 30 fs with an initial height of 1.2 kcal/mol and widths of 0.035, 0.030, and 0.020 collective variable units (c.v.u.) for qx/Q, qy/Q, and qz/Q, respectively. The simulation was extended until the FEL exhibited stability, reaching a total duration of 200 ps (6000 added potentials).

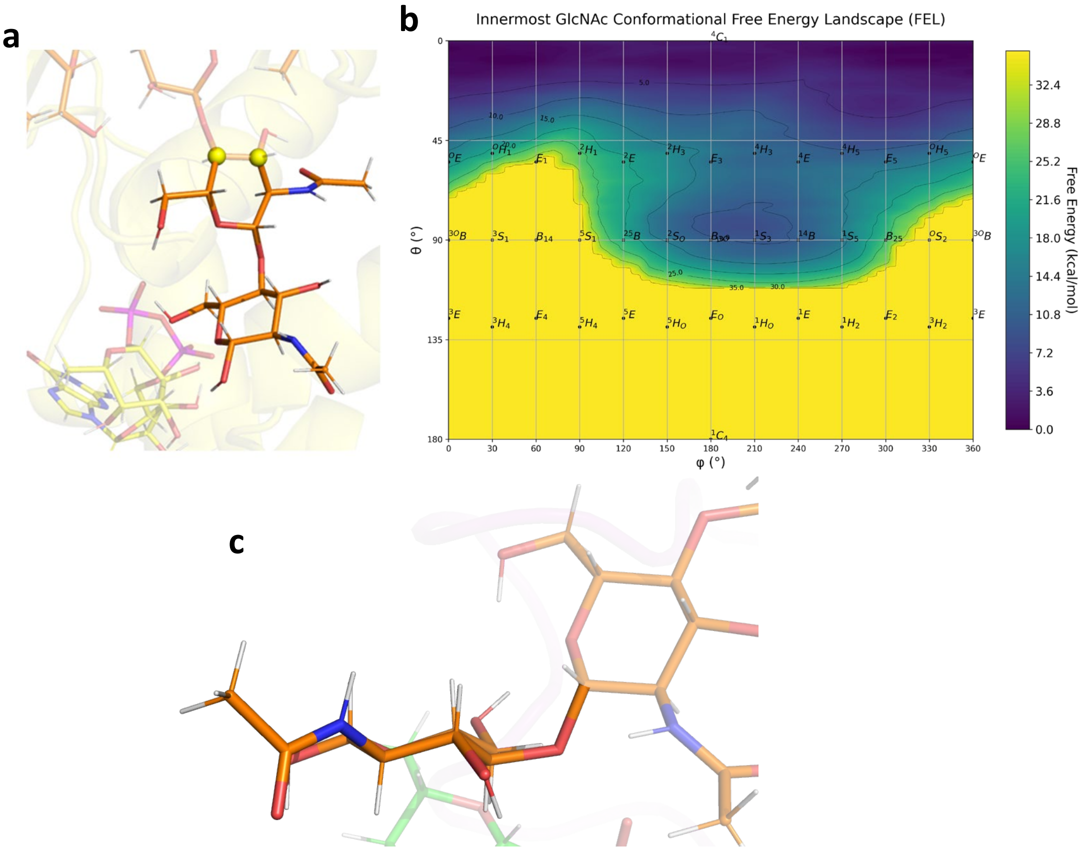

Figure S10. Metadynamics calculations. (a) QM region (solid colour) used in the QM/MM simulations for the innermost GlcNAc free energy landscape metadynamics. Yellow spheres correspond to the capping hydrogen atoms. The protein is shown in yellow, with loop 1 in white, loop 2 in purple, GDP-Fuc in green, G0 in orange, G0-Fuc in pink, and GDP-H in gray. (b) Free Energy Landscape (FEL) Analysis of innermost GlcNAc within the MC. The lowest-energy regions indicate the preferred conformations. (c) Structural representation of the innermost GlcNAc adopting the $^4C_1$ conformation (orange). The protein is shown in yellow, with loop 1 in white, loop 2 in purple, GDP-Fuc in green, and G0 in orange.

4.2.3 Fucose Transfer Free Energy Surface (FES)

For the study of sugar transfer using metadynamics, two collective variables (CV1 and CV2) were employed, each defined as the difference between two key distances. CV1 describes the fucose transfer process and is defined as the difference between the distance from the anomeric carbon of fucose to the phosphate oxygen and the distance from C1 of fucose to the oxygen of the OH6 group in the A subunit of G0, which acts as the nucleophile: $CV1 = d_{(C1-O2)} - d_{(C1-O3)}$. CV2 describes the proton transfer and is calculated as the difference between the distance from O3 to its associated proton (H6) and the distance between the carboxylate oxygen of Glu373 and the same proton: $CV2 = d_{(O3-H6)} - d_{(H6-O5)}$. The QM region included 126 atoms, confined within an orthorhombic box with dimensions of 24.1491 × 20.1220 × 19.4641 Å. These atoms correspond to those previously described in the QM/MM studies, with the addition of

the Gln470 side chain. To complete the QM region, hydrogen atoms were added for capping. A plane-wave cutoff of 330 Ry was employed. Gaussian potentials were deposited every 100 MD steps, with an initial height of 1.0 kcal/mol and a width of 0.2 for both CVs. To improve accuracy, the Gaussian height was decreased to 0.1 kcal/mol in the region near the TS. Following literature recommendations,[40] the simulation was stopped after TS recrossing, resulting in the addition of 600 Gaussian functions, with a total duration of 30 ps. The trajectory analysis was performed using PLUMED. The calculated free energy for the glycosylation reaction was compared with experimental values using transition state theory and the Eyring-Polanyi equation:

$$k = \frac{k_B T}{h} e^{-\frac{\Delta G^{\ddagger}}{RT}}$$

Where k is the experimental rate constant, and kB, h, and R are the Boltzmann, Planck, and gas constants, respectively.

The free energy surface (FES) corresponding to the fucose transfer reaction was reconstructed from the HILLS file generated during the metadynamics simulations. The reconstruction was performed using the sum_hills utility implemented in PLUMED2. To achieve a finer and more detailed description of the potential energy surface, the grid spacing for both collective variables (CVs) was customized to 0.001 units. This high-resolution grid facilitated a more precise localization of the minimum energy path (MEP) along the FES. In fact, the potential energy surface (FES) obtained from the metadynamics simulations was further analyzed using the MEPSAnd tool to perform a topological characterization.[41] This allowed the precise identification of the collective variable (CV) values associated with the reactant (RE), transition state (TS), and product (PR) structures. Moreover, the minimum energy path (MEP) connecting RE to PR via the TS was determined as the trajectory that follows the lowest free energy through the CV1–CV2 space. Using the CV values along the MEP as reference, representative structures were extracted from the full metadynamics trajectory, resulting in 300 snapshots that accurately describe the minimum energy pathway. Specific structures corresponding to desired values of key variables were located by tracking their evolution during the metadynamics simulation. Once the simulation time associated with the target values was identified, the corresponding frames were extracted from the trajectory file using CPPTRAJ. [15] Coordinates of optimized **RE_{meta},** **TS_{meta}** and **PR_{meta}** are provided as pdb files and they are illustrated in Figure S11.

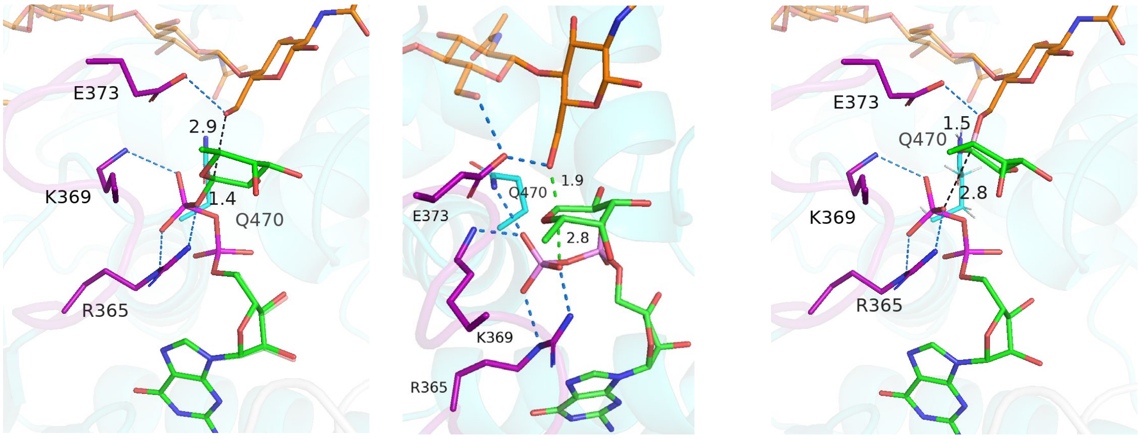

RE$_{meta}$ **TS$_{meta}$** **PR$_{meta}$**

Figure S11. Keypoints of the reaction (reactant **RE$_{meta}$**, transition structure **TS$_{meta}$** and product **PR$_{meta}$**. Geometries optimized at bp86/def2svp level of theory.

The transition-state nature of **TS$_{meta}$** was corroborated through a committor analysis,[42] in which numerous unbiased QM/MM/MD simulations were initiated from the **TS$_{meta}$** structure with randomized initial velocities. These trajectories collapsed almost indistinctly toward **RE$_{meta}$** and **PR$_{meta}$**, confirming the validity of **TS$_{meta}$** as a true transition structure.

Interestingly, during the analysis of the FES a H-transfer between Lys369 and Glu373 After Glycosylation. Structural representation (Figure S12) showing the proximity between deprotonated Lys369 and protonated Glu373 after the transfer reaction suggests a spatial arrangement leading to a potential acid-base equilibrium between these residues, which could restore the protonation state of FUT8 in its active form.

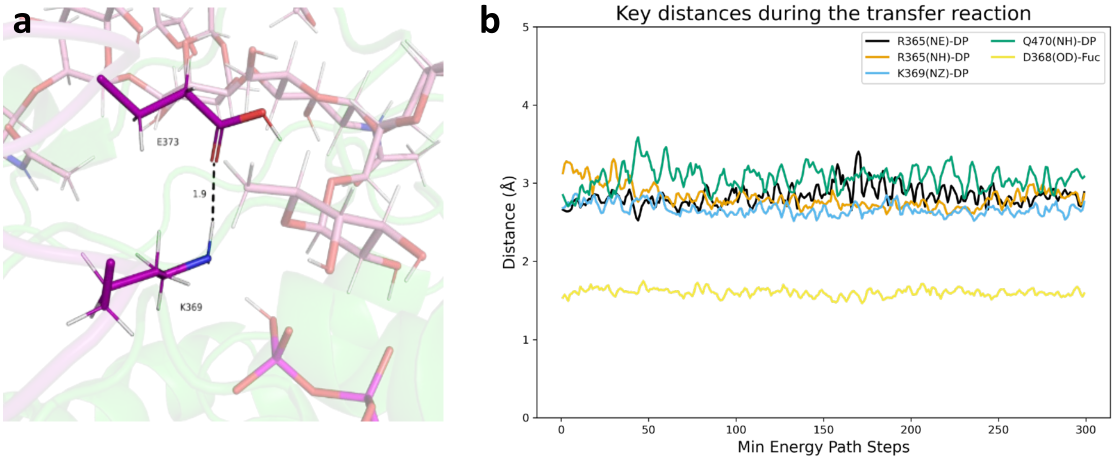

Figure S12. (a) Proton Transfer Between Lys369 and Glu373 after glycosylation. The protein is shown in yellow, with loop 1 in white, loop 2 in purple, GDP-Fuc in green, G0 in orange, G0-Fuc in pink, and GDP-H in gray. (b) Variation of key stabilizing distances along the minimum energy path. Note that the measured distances between the diphosphate (DP) and the surrounding residues refer to the bonded heavy atoms, not to the proton, which explains the slightly higher values.

The evolution of key interatomic distances involved in the stabilization of the GDP moiety as a leaving group was monitored along the 300 points representing the minimum energy path (MEP). As shown in Figure S12b, these stabilizing interactions, mainly involving residues Arg365, Lys369, D368 and Gln470, remain consistently maintained throughout the reaction coordinate. The persistence of these interactions highlights their critical role in stabilizing the transition state and facilitating efficient catalysis.

The evolution of CV1 and CV2 values throughout the metadynamics simulation was monitored to assess the progress of the reaction. As shown in Figure S13a, a clear crossing from the reactant to the product state, followed by a recrossing from product back to reactant, was observed, confirming proper sampling of the transition state region. For comparison, Figure S14b presents the variation of the four key distances involved in the fucose transfer reaction along the metadynamics simulation. The simultaneous evolution of these distances further supports the occurrence of the chemical event and highlights the consistency between the CVs behavior and the underlying bond reorganization process.

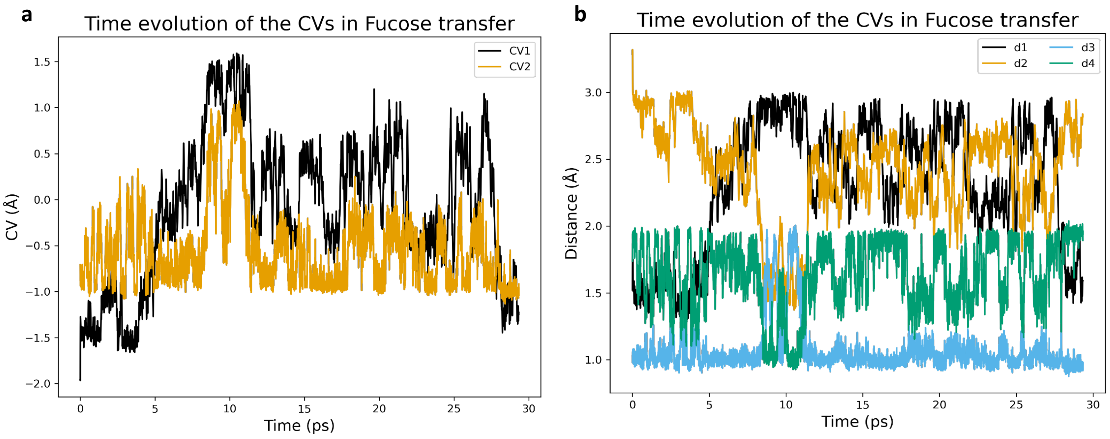

Figure S13. (a) Time evolution of the CV employed in the QM/MM metadynamics of the fucose transfer. (b) Time evolution of the key reaction distances in the QM/MM metadynamics of the fucose transfer.

4.3 Conformational behavior of involved sugar units

To characterize the conformational behavior of both sugars, fucose and the innermost GlcNAc, the Cremer–Pople puckering coordinates (θ and ϕ) were calculated at each point along the minimum free energy path. The conformations were then represented on a Mercator projection, providing a detailed visualization of the structural evolution throughout the transfer process (Figure S14).

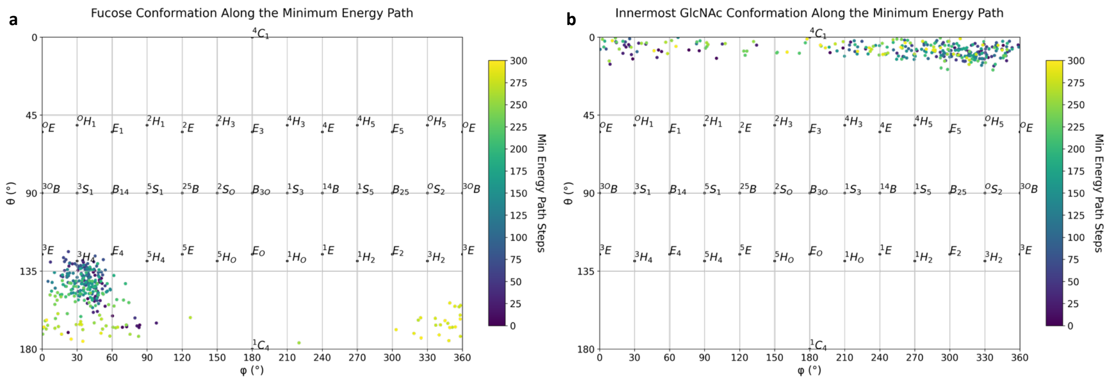

Figure S14**.** Conformational Analysis of Innermost GlcNAc and Fucose Along the Reaction Path. Free energy surface (FES) plots illustrating the conformational preferences of innermost GlcNAc and fucose during the glycosylation reaction. Throughout the simulation, the innermost GlcNAc remains in a $^4C_1$ chair conformation, while fucose adopts $^3H_4$-like conformations.

4.4 The catalytic cycle concerning the fucose transfer

The catalytic cycle considering L-fucose transfer from GDP-fucose to G(0) is illustrated in Figure S15. It can be considered a closed cycle because no external agents are involved in either the atomic balance of the reaction or in any of the steps. In fact, starting from the enzyme in its natural protonation state, we arrive to a species (**PR$_{meta}$**) in which the enzyme is in a different protonation state. Regeneration of the enzyme in its natural protonation state only requires an intramolecular acid-base equilibrium (from Glu373 to Lys369).

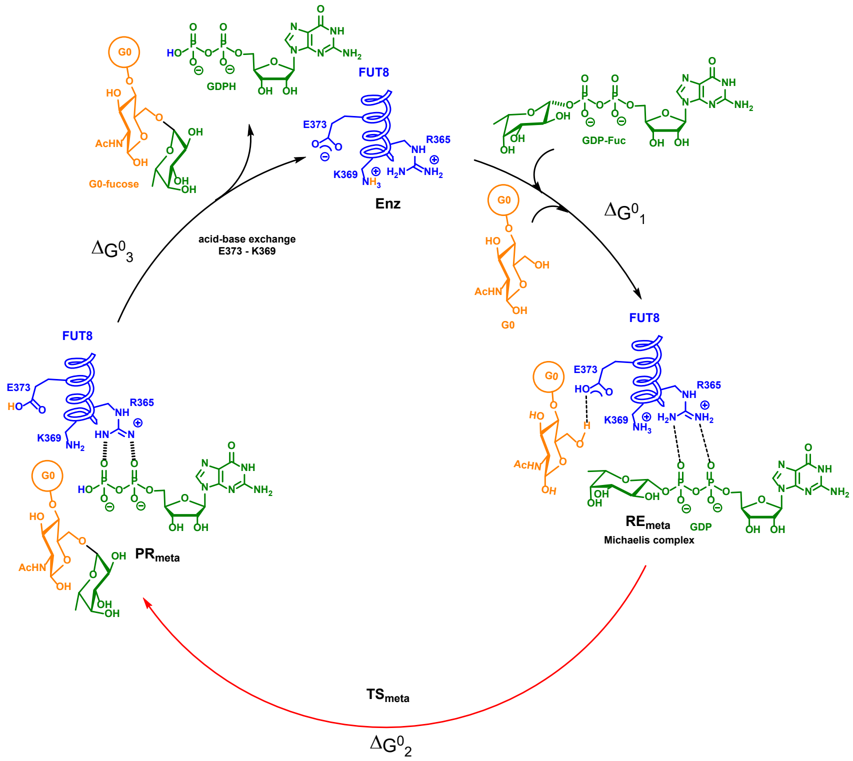

Figure S15. Catalytic cycle for L-fucose transfer from GDP-fucose to G(0).

In a closed cycle, where the initial and final state of the enzyme is the same, the total ΔG° of the cycle corresponds to the ΔG° of the net transformation of the substrate into product:

The total free energy of the cycle is:

$$\Delta G^0_{total} = \Delta G^0_1 + \Delta G^0_2 + \Delta G^0_3$$

where:

$$\Delta G^0_1 = G_{REmeta} - (G_{Enz} + G_{G0} + G_{GDP\text{-}fuc})$$

$$\Delta G^0_2 = G_{PRmeta} - G_{REmeta}$$

$$\Delta G^0_3 = (G_{Enz} + G_{GDPH} + G_{G(0)\text{-}fuc}) - G_{PRmeta}$$

Accordingly:

$$\Delta G^0_{total} = G_{REmeta} - (G_{Enz} + G_{G0} + G_{GDP\text{-}fuc}) + (G_{PRmeta} - G_{REmeta}) + (G_{Enz} + G_{GDPH} + G_{G(0)\text{-}fuc}) - - G_{PRmeta}$$

$$\Delta G^0_{total} = G_{REmeta} - G_{Enz} - G_{G0} - G_{GDP\text{-}fuc} + G_{PRmeta} - G_{REmeta} + G_{Enz} + G_{GDPH} + G_{G(0)\text{-}fuc} - G_{PRmeta}$$

Cancelling:

$$\Delta G^0_{total} = - G_{G0} - G_{GDP\text{-}fuc} + G_{GDPH} + G_{G(0)\text{-}fuc}$$

That is:

$$\Delta G^0_{total} = (G_{GDPH} + G_{G(0)\text{-}fuc}) - (G_{G0} + G_{GDP\text{-}fuc})$$

So, we calculated the free energies of reactants and products (Table S3) at the same level of the metadynamics calculations and obtained:

$$\Delta G^0_{total} = -3.4 \text{ kcal/mol}$$

Table S3. Calculated (pbepbe/(TZVP/smd=water) absolute (hartree) energies

	E(0)	G
**G(0)**	-858.394416	-858.452952
**GDP-fuc**	-2706.924467	-2707.019576
**GDPH**	-2171.939696	-2172.016246
**G(0)-fuc**	-1393.381203	-1393.461726

5 Topological calculations (ELF & NCI)

5.1 NCI (Non-Covalent Interactions)

NCI (non-covalent interactions) were computed using the methodology previously described.[43] Semi-quantitative data were obtained with the NCIPLOT4 program.[44] A density cutoff of ρ=0.5 a.u. was applied and isosurfaces of s(r) =0.32 were colored by *sign*(λ_2)ρ in the [-0.03,0.03] a.u. range using VMD software.[45] s(**r**) against *sign*(λ_2)ρ(**r**) plots were generated with gnuplot software.[16]

5.2 ELF (Electron Localization Function)

5.2.1 ELF analysis for the minimum energy path

The electronic structures of the 300 points extracted from the minimum free energy path from metadynamics calculations, as described above, were analyzed by the topological analysis of the gradient field of electron localization function (ELF) developed by Silvi and Savin.[46] The topological analysis of the gradient field of ELF has showed to be a powerful tool for the study of the bonding changes along an organic reaction[47] but it has scarcely been used for enzymatic reactions.[48]

The generation of the electron density (density.cube) and electron localization function (elf.cube) cube files required for the ELF analysis was carried out for each one of the 300 points extracted from the minimum free energy path, using the corresponding modules implemented in CP2K. Specifically, the E_DENSITY_CUBE and ELF_CUBE options within the FORCE_EVAL/DFT/PRINT section of the CP2K input were employed. Both properties

were printed with a stride of 1 1 1, ensuring maximum resolution of the cube files. The obtained cube files for each point were processed with the Multiwfn software[49] to obtain the corresponding .out files with information on basins and population and .gjf files with the attractors positions. These data were used for composing the Figure S16.

For that analysis we use the following Multiwfn options:

```
17      // Basin analysis
1       // Generate basins and locate attractors
2       // Medium quality grid
-4, 4   // Export attractors as gjf file      # generate the .gjf file
2, -1   // Topology analysis                   # generate the .out file
```

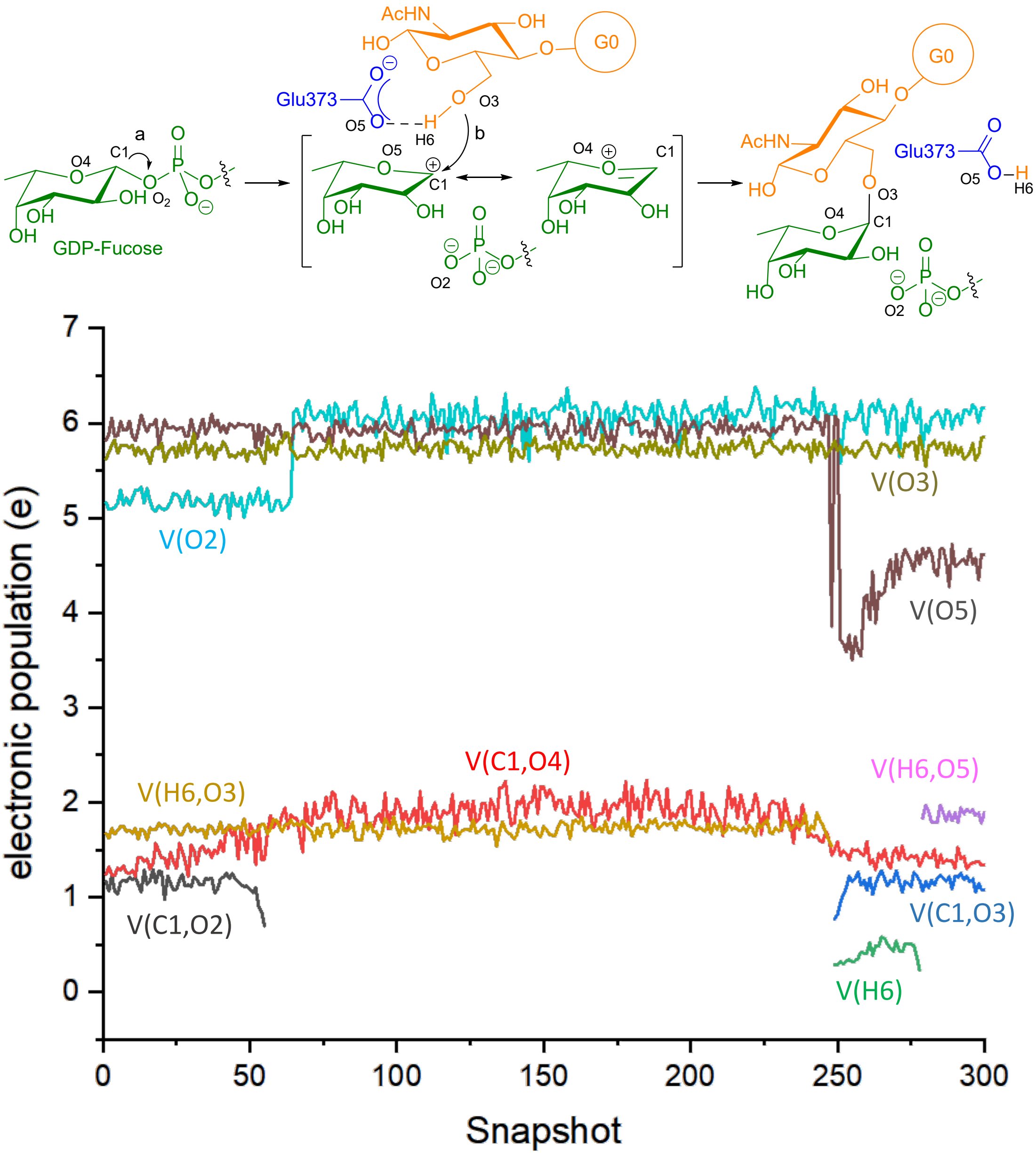

Figure S16. ELF analysis for the fucosylation of the reaction (QM section). The different traces represent the evolution of the electronic population in a similar way to curve arrows indicate the movement of electrons.

Table S4. ELF basin populations for the fucosylation reaction represented in Figure 16.

	V(C1,O2)	V(C1,O5)	V(C1,O3)	V(H6)	V(H6,O5)	V(H6,O3)	V(O2)	V(O5)	V(O4)
1	1.16437	1.24039				1.67593	5.1651	5.82374	5.62009
2	1.06639	1.32048				1.62295	5.09373	5.88457	5.69259
3	1.23049	1.23323				1.72609	5.09457	6.05114	5.76604
4	1.06604	1.22846				1.66761	5.11198	5.86022	5.82117
5	1.03244	1.30777				1.70494	5.15724	5.87018	5.68314
6	1.15843	1.28586				1.7448	5.29445	6.0159	5.76123
7	1.18124	1.33091				1.7527	5.14091	5.92337	5.76855
8	1.16503	1.26122				1.69149	5.21303	5.92338	5.76536
9	1.21064	1.24988				1.70682	5.13813	6.02059	5.74066
10	1.18196	1.28279				1.67496	5.20533	5.86953	5.76653

11	1.2127	1.22268				1.72734	5.29233	5.81547	5.65815
12	1.13341	1.42082				1.61572	5.29491	5.92574	5.74667
13	1.05619	1.42208				1.72091	5.32543	6.08452	5.67837
14	1.28402	1.37725				1.6962	5.15744	5.98549	5.72122
15	1.1347	1.40297				1.60559	5.31273	5.90975	5.67002
16	1.17262	1.29818				1.73327	5.17606	5.98596	5.57527
17	1.29048	1.35294				1.70883	5.08831	5.98179	5.70602
18	1.15208	1.56766				1.71607	5.07183	6.03762	5.74307
19	1.274	1.48269				1.78161	5.12972	5.77149	5.73313
20	1.26874	1.41686				1.74167	5.09645	5.89915	5.78916
21	0.97461	1.40977				1.76852	5.08655	6.03658	5.59454
22	1.22464	1.304				1.74959	5.11363	5.95229	5.67257
23	1.14711	1.47237				1.67486	5.16693	5.9508	5.72614
24	1.07528	1.37786				1.75173	5.25617	5.96343	5.74641
25	1.08235	1.30319				1.70939	5.25105	5.92872	5.63987
26	1.17843	1.56978				1.66413	5.22377	5.94739	5.78251
27	1.23818	1.4796				1.6652	5.11271	5.95432	5.66192
28	1.11774	1.37959				1.73934	5.21673	5.86193	5.80362
29	1.1311	1.22087				1.77138	5.28406	5.86714	5.68898
30	1.10933	1.5489				1.69485	5.12629	6.04274	5.78046
31	1.21546	1.49447				1.73756	5.127	5.91503	5.89159
32	1.18501	1.34914				1.69284	5.21176	5.84109	5.63587
33	1.13264	1.50475				1.70979	5.18657	5.94544	5.71158
34	1.12741	1.53982				1.71299	5.14268	6.0439	5.72719
35	1.19898	1.46806				1.6222	5.12642	5.88176	5.78548
36	1.19034	1.50171				1.75697	5.1594	5.94888	5.67378
37	1.20217	1.42029				1.74018	5.25409	6.09595	5.76146
38	1.04507	1.49839				1.67281	5.03143	5.96562	5.62064
39	1.08457	1.46388				1.69317	5.05297	6.02939	5.72929
40	1.19405	1.59378				1.79473	5.17285	5.90492	5.72008
41	1.21029	1.4827				1.67778	5.22914	5.97404	5.80228
42	1.25244	1.72666				1.69919	5.24012	5.89656	5.74707
43	1.19703	1.72974				1.76279	4.99731	5.99153	5.64512
44	1.25272	1.65464				1.73491	5.26411	5.9791	5.66421
45	1.19424	1.71086				1.71053	5.14252	5.95265	5.63571
46	1.15985	1.40279				1.74901	5.12352	5.99262	5.68649
47	1.13446	1.72492				1.71378	5.21113	5.98442	5.66588
48	1.11871	1.59711				1.82595	5.00668	5.98452	5.72417
49	1.12458	1.47004				1.67895	5.12514	5.95773	5.59965
50	1.08244	1.77336				1.64532	5.25681	5.98672	5.70259
51	1.05298	1.7095				1.69898	5.14387	5.97727	5.693
52	1.19178	1.34717				1.69697	5.21679	5.73659	5.67265
53	0.90017	1.75727				1.71222	5.31652	5.98454	5.72655
54	0.85218	1.40135				1.77133	5.1339	5.76831	5.64336
55	0.78127	1.40231				1.76455	5.17432	5.95356	5.57307
56		1.68724				1.66939	5.27897	5.92341	5.85992
57		1.86136				1.72033	5.06974	5.88238	5.69667
58		1.74037				1.8202	5.0817	5.87483	5.77202
59		1.80939				1.81173	5.18058	5.9846	5.6696
60		1.75067				1.75305	5.18203	5.98457	5.81008
61		1.86845				1.69175	5.08179	5.84023	5.78766
62		1.94257				1.76664	5.10876	5.84121	5.89873
63		1.72739				1.76831	5.14514	5.88086	5.89809
64		1.86962				1.81014	5.22204	5.76098	5.71628
65		1.84011				1.76318	6.10886	5.946	5.69495

66		1.8272				1.7784	6.15417	5.98481	5.62293
67		1.64473				1.75098	6.06699	5.99779	5.65249
68		1.47708				1.80601	5.92015	5.94252	5.65742
69		1.87881				1.75656	6.17472	5.99374	5.65926
70		1.78767				1.72273	6.2391	5.9501	5.74134
71		1.76317				1.69549	6.08705	5.80385	5.71569
72		2.00308				1.59765	6.03378	5.82721	5.65936
73		1.8395				1.73044	6.07143	5.88031	5.61621
74		1.83653				1.64381	6.12547	5.88304	5.71279
75		1.90281				1.75827	6.1121	5.81793	5.6985
76		2.05226				1.77049	6.03002	5.80943	5.73745
77		1.91355				1.76942	6.1836	5.94675	5.83886
78		2.11032				1.6341	5.96528	5.76289	5.69132
79		2.08428				1.69246	6.21099	6.02532	5.73005
80		1.79709				1.69298	6.14825	6.01391	5.67075
81		1.91498				1.81812	5.9642	5.9056	5.62418
82		1.71695				1.70444	6.09957	6.03054	5.81794
83		1.72519				1.69887	5.9572	5.93744	5.66979
84		1.84954				1.72247	6.16963	5.86771	5.72618
85		2.06428				1.79236	6.139	5.93918	5.68429
86		2.03134				1.608	6.22937	5.90913	5.63887
87		1.89836				1.601	5.97708	5.87571	5.7561
88		1.89836				1.601	5.97708	5.87571	5.7781
89		1.99414				1.77237	6.0393	5.97602	5.59255
90		1.7406				1.70624	6.08033	5.90094	5.73335
91		1.77331				1.78295	6.04821	5.95747	5.70254
92		1.741				1.69377	6.03283	5.90276	5.7191
93		1.84747				1.69721	5.85355	6.02041	5.68263
94		1.8795				1.6088	6.04315	5.90898	5.79199
95		1.78939				1.67533	6.07578	5.95377	5.60885
96		2.10053				1.82788	6.26883	6.014	5.90478
97		1.72363				1.77185	6.12522	5.93641	5.80776
98		1.89349				1.72589	5.96742	5.92044	5.7428
99		1.86489				1.84752	5.94156	5.89878	5.78015
100		2.03763				1.73033	6.02409	5.88272	5.71746
101		1.95632				1.65399	6.18225	5.85989	5.79047
102		1.80006				1.79672	5.99501	5.92716	5.74599
103		1.90385				1.67746	5.9308	5.82976	5.90629
104		1.78441				1.72207	6.00521	5.959	5.77685
105		1.84919				1.69539	5.90904	6.05131	5.71708
106		1.9361				1.68968	6.12645	5.93291	5.68064
107		1.75081				1.75532	6.03423	5.70939	5.7851
108		2.11773				1.71239	6.0476	5.75659	5.79552
109		1.78165				1.75098	6.10674	5.85507	5.71861
110		1.90053				1.75093	6.06219	5.88424	5.76577
111		1.71299				1.77158	5.90675	5.88486	5.64709
112		1.91731				1.7347	6.30957	6.06814	5.72107
113		1.99665				1.7423	6.15631	5.88736	5.76886
114		1.83778				1.6832	5.98598	5.94852	5.79226
115		1.83836				1.79954	6.1406	5.92345	5.62696
116		1.91087				1.5908	6.13096	5.96921	5.80472
117		1.88335				1.63421	5.68117	5.90788	5.77947
118		1.89984				1.60713	6.19921	5.92663	5.81826
119		2.0859				1.69002	5.86126	5.9558	5.80945
120		1.65608				1.7513	6.17858	5.93462	5.79447

121		1.8997				1.65312	6.20475	5.90093	5.70137
122		2.00481				1.69764	6.01007	5.96915	5.80092
123		1.93683				1.71175	6.16598	5.88254	5.69193
124		2.01271				1.71865	6.11993	5.91594	5.74026
125		1.81757				1.70079	5.84108	5.91738	5.5852
126		2.01585				1.81576	6.02458	5.94768	5.69782
127		1.79396				1.68144	6.26713	5.95554	5.67142
128		1.81858				1.6943	6.00614	5.97178	5.66449
129		1.99013				1.72168	6.05514	5.78269	5.79747
130		1.99001				1.75995	6.06521	5.94396	5.86719
131		1.80983				1.66835	6.04234	5.83846	5.79147
132		2.03145				1.70688	6.02923	6.06654	5.65587
133		1.96399				1.71147	6.06146	6.05133	5.80979
134		1.81465				1.54103	5.95932	5.89055	5.69314
135		2.14946				1.69284	5.93887	5.91815	5.74505
136		2.1586				1.79392	6.06766	5.9596	5.80686
137		2.22269				1.70856	6.31119	6.08461	5.84435
138		1.93975				1.6821	5.96675	5.88754	5.82768
139		1.76165				1.65425	5.75527	5.78492	5.60257
140		1.78633				1.61772	6.12059	5.92233	5.72655
141		2.0118				1.6659	6.05347	5.97384	5.71132
142		1.98251				1.80503	6.19558	5.93686	5.64386
143		2.06416				1.72125	5.72263	6.02237	5.63411
144		1.84046				1.78402	6.1051	5.9219	5.78859
145		1.99533				1.69084	5.60313	6.04781	5.76071
146		2.01134				1.70748	6.14411	5.98184	5.7683
147		2.17253				1.73806	6.1537	5.97641	5.85431
148		1.88302				1.66305	6.05417	5.98576	5.7009
149		2.17019				1.63792	6.08937	5.97043	5.73111
150		2.16195				1.74591	6.10803	6.01393	5.75706
151		2.05525				1.71003	5.88759	5.97498	5.73706
152		2.13055				1.66568	6.11594	5.83064	5.76979
153		2.07907				1.65267	6.14017	5.89699	5.71248
154		1.92273				1.65176	6.18029	5.99439	5.79924
155		2.02151				1.74836	6.1447	5.89172	5.62188
156		2.0163				1.80313	6.25954	5.80995	5.79801
157		1.89336				1.77922	6.13002	5.91279	5.80518
158		1.88031				1.76138	6.36872	5.89863	5.72903
159		2.06038				1.62053	6.16255	6.10269	5.84345
160		1.91718				1.80389	6.16577	6.07781	5.74221
161		1.87731				1.80884	6.18289	5.88625	5.71758
162		1.84217				1.77857	6.09056	5.91865	5.77934
163		2.10721				1.85039	5.79067	5.96955	5.75487
164		1.86622				1.70381	6.11443	5.87341	5.75994
165		2.09706				1.73736	6.03661	6.05207	5.76561
166		1.89616				1.75637	6.00089	5.83538	5.74346
167		1.70171				1.69809	6.21414	5.82299	5.67238
168		1.86537				1.70993	6.21632	5.93711	5.74105
169		1.87711				1.73688	6.14971	6.03582	5.71586
170		1.88431				1.68919	5.79597	5.98864	5.87629
171		2.01993				1.79772	6.1128	5.89326	5.66574
172		1.8153				1.68788	6.15465	5.78176	5.69478
173		1.95318				1.68031	5.85636	5.92366	5.73991
174		1.98905				1.77686	6.1246	5.7484	5.70286
175		1.93509				1.75703	6.01776	5.93047	5.64218

176		2.03251				1.71845	6.09787	6.04548	5.66773
177		2.00253				1.83462	6.10758	6.04722	5.76944
178		2.21875				1.71728	6.22212	5.98536	5.74514
179		1.74836				1.78952	6.08176	5.97899	5.72888
180		2.16765				1.64262	5.88453	5.99362	5.68237
181		1.92068				1.7098	6.16752	6.0084	5.7366
182		2.04027				1.76019	6.15426	6.01954	5.64551
183		1.79683				1.72236	6.14665	5.8635	5.77244
184		2.03914				1.73723	6.06776	5.90846	5.77827
185		2.2328				1.69329	6.03426	5.92195	5.68749
186		1.92879				1.8168	6.10204	5.85087	5.81046
187		2.0285				1.78094	6.2062	5.91289	5.7196
188		2.1176				1.82127	5.9686	5.84297	5.80618
189		2.10422				1.72556	6.23848	6.07017	5.67878
190		1.94079				1.75331	6.16294	5.95051	5.75165
191		1.84885				1.78626	6.04819	5.90557	5.66505
192		2.13878				1.75731	6.03423	5.79392	5.72839
193		1.82383				1.77673	5.94015	5.96566	5.64989
194		1.88601				1.77501	6.17104	5.8023	5.76554
195		1.83674				1.71033	6.07408	5.79798	5.77047
196		1.93115				1.80082	5.67944	5.91706	5.64583
197		1.97199				1.76291	6.10915	5.94604	5.77709
198		1.85484				1.80955	6.23129	6.01622	5.92944
199		2.10852				1.76135	6.13053	5.89432	5.68852
200		2.12072				1.74765	6.08384	5.96459	5.6575
201		1.75496				1.74104	5.95725	6.0254	5.7033
202		1.87055				1.71001	6.07986	5.9512	5.64053
203		1.85413				1.7378	5.85934	5.84063	5.7997
204		2.10383				1.71589	6.11999	5.97493	5.74912
205		1.8771				1.69693	6.13527	5.99371	5.64648
206		1.71292				1.77242	6.13181	6.01315	5.84412
207		1.98328				1.67325	6.186	5.96674	5.78685
208		1.94587				1.77787	6.02006	5.96225	5.68252
209		1.85212				1.7135	6.13295	5.88256	5.62695
210		1.79885				1.77911	6.19869	6.03351	5.76045
211		1.65203				1.66972	6.0802	5.89974	5.69982
212		1.88451				1.69659	6.27095	6.07756	5.77527
213		2.16374				1.76936	6.02594	5.88878	5.67264
214		1.94419				1.7147	6.10357	5.96998	5.81186
215		2.04666				1.73958	5.97103	5.92744	5.71729
216		1.83388				1.7148	6.13247	5.91255	5.76083
217		2.08031				1.69888	6.13796	5.97764	5.77999
218		1.97486				1.73594	6.08085	5.87259	5.84215
219		1.78122				1.68137	6.07244	5.95724	5.73515
220		1.91029				1.76157	6.13037	5.93095	5.86401
221		1.91368				1.72095	6.22416	5.99615	5.76348
222		2.09099				1.64615	6.37803	5.91234	5.64312
223		1.99027				1.77315	6.32705	6.00076	5.6774
224		1.66178				1.73424	6.15962	5.91262	5.68724
225		1.8866				1.78877	6.02616	5.9315	5.79622
226		1.76501				1.82035	6.14527	5.907	5.78518
227		1.97778				1.68445	6.15089	5.86284	5.83225
228		2.01537				1.67012	5.83775	5.95264	5.7645
229		1.98436				1.73372	6.27954	5.89904	5.74274
230		1.98328				1.6943	6.179	5.96148	5.71279

231		1.70577				1.72592	6.16865	5.93629	5.67461
232		2.03301				1.80691	6.20779	6.06908	5.75451
233		1.90554				1.79037	6.21329	6.03133	5.7337
234		1.78759				1.71174	6.18354	6.0674	5.81332
235		1.57244				1.74627	6.1428	6.0449	5.70175
236		1.8535				1.80157	6.24262	5.88487	5.76304
237		1.71845				1.74289	6.24974	5.97146	5.77169
238		1.97531				1.77505	6.09994	6.00648	5.73237
239		1.8532				1.8816	6.15241	5.95022	5.71418
240		1.63424				1.66983	6.11599	5.93424	5.77152
241		1.66846				1.75864	6.09471	5.9355	5.59104
242		1.63881				1.72041	6.3675	6.06783	5.82508
243		1.64667				1.89055	6.00408	6.00346	5.66967
244		1.59216				1.81238	6.21874	6.00832	5.79835
245		1.58508				1.76511	6.21094	5.87621	5.74958
246		1.69712				1.68711	6.09806	6.07869	5.63782
247		1.63132				1.60118	6.0978	6.07721	5.695
248		1.44116				1.54167	6.09186	3.85838	5.74413
249		1.49938	0.77261	0.28889			6.19308	6.07676	5.8128
250		1.57932	0.82178	0.29887			5.96911	6.04364	5.7086
251		1.44133	0.94161	0.31663			5.5794	3.71035	5.75776
252		1.49268	1.03002	0.29361			5.91132	3.67674	5.66648
253		1.46019	1.09666	0.32623			6.11716	3.58915	5.74609
254		1.39396	1.22024	0.34122			6.03154	3.76048	5.68357
255		1.50371	1.16588	0.32221			6.03594	3.50253	5.70726
256		1.49131	1.1973	0.33099			6.03872	3.73962	5.64636
257		1.5029	1.26525	0.3534			6.09649	3.59669	5.70441
258		1.45837	1.12749	0.41906			6.23121	3.54555	5.72151
259		1.44256	1.181	0.41093			5.94305	4.16148	5.79708
260		1.58726	1.07388	0.41019			5.88541	4.18882	5.80692
261		1.35526	1.20382	0.51951			6.17312	3.92618	5.7547
262		1.39469	1.02968	0.38985			6.20042	4.43248	5.6708
263		1.29141	1.14473	0.4888			6.11816	3.89145	5.63196
264		1.45865	1.22447	0.48123			6.24506	4.21903	5.6958
265		1.40127	1.28155	0.58183			6.1308	4.18332	5.78158
266		1.5034	1.19826	0.56442			6.13331	4.22618	5.77599
267		1.36455	1.12044	0.50301			5.88365	4.43415	5.75013
268		1.30528	1.17746	0.46362			6.08868	4.56045	5.68804
269		1.46861	1.16163	0.4313			6.18875	4.26674	5.74121
270		1.5172	1.27961	0.53489			6.12207	4.61656	5.78807
271		1.39495	1.17167	0.43323			5.64803	4.35514	5.69407
272		1.54233	1.05386	0.42507			6.04532	4.57436	5.64643
273		1.41876	1.1654	0.50116			5.91515	4.66246	5.78923
274		1.40162	1.14836	0.5014			6.18015	4.60432	5.75065
275		1.44186	1.11187	0.5095			6.24534	4.53345	5.79511
276		1.42146	1.2635	0.48972			6.07681	4.44365	5.80043
277		1.43669	1.22057	0.37605			6.00095	4.64113	5.6209
278		1.32724	1.16956	0.2311			6.15878	4.51241	5.86601
279		1.40864	1.06742		1.83555		5.74859	4.68001	5.719
280		1.47708	1.16568		1.96767		5.98256	4.58342	5.54257
281		1.42114	1.09993		1.9084		5.90022	4.67936	5.7606
282		1.41573	1.18006		1.79825		6.05457	4.63035	5.76687
283		1.50075	1.1309		1.7886		6.06911	4.4438	5.81276
284		1.31422	1.20468		1.79407		6.12181	4.39012	5.72909
285		1.4457	1.23333		1.9683		6.05984	4.67135	5.7815

286		1.36126	1.20135		1.8923		5.95077	4.67392	5.79099
287		1.39397	1.16737		1.80383		6.1398	4.53563	5.66254
288		1.38584	1.21851		1.9012		6.11674	4.27296	5.79453
289		1.38459	1.0899		1.80949		5.94133	4.72169	5.66284
290		1.30842	1.22397		1.88719		6.06025	4.48714	5.76306
291		1.52408	1.21236		1.78961		6.09266	4.5138	5.67694
292		1.40466	1.25236		1.94032		5.98728	4.54415	5.73923
293		1.42988	1.1875		1.87886		6.13365	4.56384	5.62142
294		1.30201	1.22026		1.92488		6.13479	4.51098	5.6462
295		1.34316	1.16511		1.89973		6.09446	4.67046	5.68796
296		1.36692	1.19299		1.92132		6.19302	4.516	5.78511
297		1.36915	1.05796		1.864		6.15156	4.57398	5.61354
298		1.3785	1.14581		1.89107		6.01091	4.34762	5.66562
299		1.33699	1.08184		1.78486		6.15684	4.61174	5.84611
300		1.33699	1.07166		1.89048		6.15684	4.61174	5.85233

5.2.2 ELF analyses for MD trajectories

The ELF analysis described in section 5.2.1 can be considered analogous to a conventional IRC (Intrinsic Reaction Coordinate) analysis in QM calculations. However, it provides significantly more detailed and accurate information than a simple bond-breaking/forming diagram, as it monitors the evolution of the electron density. This allows us to determine the exact moment a bond is broken or formed.

Both representations, however, unfold along the reaction coordinate and not over time. To access time-resolved information, a trajectory must be defined. To this end, we performed 20 molecular dynamics (MD) simulations starting from the transition state structure, allowing the system to evolve over time (see methods for details). Each of these partial simulations was initiated with a statistical factor and in pairs with initial velocities v and –v, such that the trajectories initially evolved in opposite directions. This approach yielded a full trajectory from reactant to product through the transition state. Performing ELF analysis on a given MD trajectory reveals the state of the reaction as a function of time. Due to the stochastic nature of MD simulations, a sufficiently large number of trajectories must be generated to achieve minimal statistical significance. Admittedly, twenty is not a sufficiently large number to be considered statistically significant in a quantitative sense; however, it is important to note the enormous computational time required to perform the QM/MM/MD dynamics and subsequently carry out an ELF analysis for each of them. However, they are, sufficient to provide a qualitative analysis of the situation, which leaves no doubt, as there is a clear gap between the breaking of one glycosidic bond and the formation of the other.

The ELF analysis of MD simulations is carried out in the same way as described above for the minimum energy path. An Excel with all the ELF basin populations and the corresponding graphics of the 20 individual MD simulations (those depicted in the Figure 7 of the main text) is available upon request.

6 Additional supporting material

Additional supplementary material is deposited at ZENODO:

https://zenodo.org/records/17496620

DOI: 10.5281/zenodo.17496620

This materials is formed by

6.1 PDB Files

MD_trajectory-01a.pdb: starting structures of a simulation of *apo form (inactive)*
MD_trajectory-01b.pdb: final structures of a simulation of *apo form (inactive)*
MD_trajectory-02a.pdb: starting structures of a simulation of *closed conformation (active)*
MD_trajectory-02b.pdb: final structures of a simulation of *closed conformation (active)*
MD_trajectory-03a.pdb: starting structures of a simulation of *closed conformation in complex with GDP-fucose*
MD_trajectory-03b.pdb: final structures of a simulation of *closed conformation in complex with GDP-fucose*
MD_trajectory-04a.pdb: starting structures of a simulation of *Michaelis complex*
MD_trajectory-04b.pdb: final structures of a simulation of *Michaelis complex*

QMMM_RE.pdb: RE_{QM}
QMMM_TS.pdb: TS_{QM}
QMMM_PR.pdb: PR_{QM}

METADYN_RE.pdb: RE_{meta}
METADYN_TS.pdb: TS_{meta}
METADYN_PR.pdb: PR_{meta}

6.2 Video Files

Descriptors-ELF.mp4

The attached video file shows the course of the 300 points extracted from the FES (minimum free energy path) as depicted by the ELF analysis. This video clearly shows the three stages of the reaction: (i) breaking of the bond between the anomeric center and the phosphate oxygen; (ii) formation of the glycosidic bond and (iii) H-transfer. Between (i) and (ii) the presence of the transient glycosyl cation is evident and it can be appreciated the planarity of the anomeric carbon. The atoms and bonds involved in the reaction are represented in tube and the rest in ball and stick. The purple spheres are the attractors generated in the ELF analysis (See main text)

Reaction.mp4

The attached video file shows the course of the 300 points extracted from the FES (minimum free energy path). This video also clearly shows the three stages of the

reaction. In fact, it is evident that for most of the reaction, the fucose ring exists as a carbocationic species, until the glycosidic bond is finally formed, at which point proton transfer to the catalytic base takes place

7 References

(1) Case, D. A.; Ben-Shalom, I. Y.; Brozell, S. R.; Cerutti, D. S.; III, T. E. C.; Cruzeiro, V. W. D.; Darden, T. A.; Duke, R. E.; Ghoreishi, D.; Gilson, M. K.; Gohlke, H.; Goetz, A. W.; Greene, D.; Harris, R.; Homeyer, N.; Huang, Y.; Izadi, S.; Kovalenko, A.; Kurtzman, T.; Lee, T. S.; LeGrand, S.; Li, P.; Lin, C.; Liu, J.; Luchko, T.; Luo, R.; Mermelstein, D. J.; Merz, K. M.; Miao, Y.; Monard, G.; Nguyen, C.; Nguyen, H.; Omelyan, I.; Onufriev, A.; Pan, F.; Qi, R.; Roe, D. R.; Roitberg, A.; Sagui, C.; Schott-Verdugo, S.; Shen, J.; Simmerling, C. L.; Smith, J.; Salomon-Ferrer, R.; Swails, J.; Walker, R. C.; Wang, J.; Wei, H.; Wolf, R. M.; Wu, X.; Xiao, L.; York, D. M.; Kollman, P. A. *AMBER 2023, University of California, San Francisco* **2023**.

(2) Maier, J. A.; Martinez, C.; Kasavajhala, K.; Wickstrom, L.; Hauser, K. E.; Simmerling, C. ff14SB: Improving the Accuracy of Protein Side Chain and Backbone Parameters from ff99SB *J. Chem. Theory Comput.* **2015**, *11*, 3696–3713.

(3) Wang, J.; Wolf, R. M.; Caldwell, J. W.; Kollman, P. A.; Case, D. A. Development and testing of a general amber force field *J. Comput. Chem.* **2004**, *25*, 1157–1174.

(4) Kirschner, K. N.; Yongye, A. B.; Tschampel, S. M.; Gonzalez-Outeirino, J.; Daniels, C. R.; Foley, B. L.; Woods, R. J. GLYCAM06: a generalizable biomolecular force field. Carbohydrates *J. Comput. Chem.* **2008**, *29*, 622–655.

(5) Price, D. J.; Brooks, C. L., III A modified TIP3P water potential for simulation with Ewald summation *The Journal of Chemical Physics* **2004**, *121*, 10096–10103.

(6) Davidchack, R. L.; Handel, R.; Tretyakov, M. V. Langevin thermostat for rigid body dynamics *J. Chem. Phys.* **2009**, *130*, 234101.

(7) Darden, T.; York, D.; Pedersen, L. Particle mesh Ewald: an N·log(N) method for Ewald sums in large systems *J. Chem. Phys.* **1993**, *98*, 10089.

(8) Ryckaert, J. P.; Ciccotti, G.; Berendsen, H. J. C. Numerical integration of the Cartesian equations of motion of a system with constraints: molecular dynamics of n-alkanes *J. Comput. Phys.* **1977**, *23*, 327.

(9) Wan, S.; Sinclair, R. C.; Coveney, P. V. Uncertainty quantification in classical molecular dynamics *Philos. Trans. A Math. Phys. Eng. Sci.* **2021**, *379*, 20200082.

(10) Garcia-Garcia, A.; Ceballos-Laita, L.; Serna, S.; Artschwager, R.; Reichardt, N. C.; Corzana, F.; Hurtado-Guerrero, R. Structural basis for substrate specificity and catalysis of α1,6-fucosyltransferase *Nat. Commun.* **2020**, *11*, 973.

(11) Pettersen, E. F.; Goddard, T. D.; Huang, C. C.; Couch, G. S.; Greenblatt, D. M.; Meng, E. C.; Ferrin, T. E. UCSF Chimera-A visualization system for exploratory research and analysis *J. Comput. Chem.* **2004**, *25*, 1605–1612.

(12) Ihara, H.; Ikeda, Y.; Toma, S.; Wang, X.; Suzuki, T.; Gu, J.; Miyoshi, E.; Tsukihara, T.; Honke, K.; Matsumoto, A.; Nakagawa, A.; Taniguchi, N. Crystal structure of mammalian α1,6-fucosyltransferase, FUT8 *Glycobiology* **2007**, *17*, 455–466.

(13) Schrodinger, L.; 3.0 ed.

(14) Olsson, M. H. M.; Sondergaard, C. R.; Rostkowski, M.; Jensen, J. H. PROPKA3: Consistent Treatment of Internal and Surface Residues in Empirical pKa Predictions *J. Chem. Theory Comput.* **2011**, *7*, 525–537.

(15) Roe, D. R.; Cheatham, T. E. PTRAJ and CPPTRAJ: Software for Processing and Analysis of Molecular Dynamics Trajectory Data *J. Chem. Theory Comput.* **2013**, *9*, 3084–3095.

(16) Williams, T.; Kelley, C. Gnuplot 4.6: an interactive plotting pogram *htto://gnuplot.sourceforge.net* **2013**.

(17) Lu, Y.; Sen, K.; Yong, C.; Gunn, D. S. D.; Purton, J. A.; Guan, J.; Desmoutier, A.; Abdul Nasir, J.; Zhang, X.; Zhu, L.; Hou, Q.; Jackson-Masters, J.; Watts, S.; Hanson, R.; Thomas, H. N.; Jayawardena, O.; Logsdail, A. J.; Woodley, S. M.; Senn, H. M.; Sherwood, P.; Catlow, C. R. A.; Sokol, A. A.; Keal, T. W. Multiscale QM/MM modelling of catalytic systems with ChemShell *Phys. CHem. Chem. Phys.* **2023**, *25*, 21816–21835..

(18) Sanz-Martinez, I.; Garcia-Garcia, A.; Tejero, T.; Hurtado-Guerrero, R.; Merino, P. The Essential Role of Water Molecules in the Reaction Mechanism of Protein O-Fucosyltransferase 2 *Angew. Chem., Int. Ed.* **2022**, *61*, e202213610

(19) Dohn, A. O. Multiscale electrostatic embedding simulations for modeling structure and dynamics of molecules in solution: A tutorial review *Int. J. Quantum Chem.* **2020**, *120*, e26343.

(20) Becke, A. D. Density-functional exchange-energy approximation with correct asymptotic behavior *Phys. Rev. A Gen. Phys.* **1988**, *38*, 3098–3100.

(21) (a) Schäfer, A.; Horn, H.; Ahlrichs, R. Fully optimized contracted Gaussian basis sets for atoms Li to Kr *J. Chem. Phys.* **1992**, *97*, 2571–2577. (b) Schäfer, A.; Huber, C.; Ahlrichs, R. Fully optimized contracted Gaussian basis sets of triple zeta valence quality for atoms Li to Kr *J. Chem. Phys.* **1994**, *100*, 5829–5835.

(22) Bakowies, D.; Thiel, W. Hybrid Models for Combined Quantum Mechanical and Molecular Mechanical Approaches *J. Phys. Chem.* **1996**, *100*, 10580–10594.

(23) Lu, Y.; Sen, K.; Yong, C.; Gunn, D. S. D.; Purton, J. A.; Guan, J.; Desmoutier, A.; Abdul Nasir, J.; Zhang, X.; Zhu, L.; Hou, Q.; Jackson-Masters, J.; Watts, S.; Hanson, R.; Thomas, H. N.; Jayawardena, O.; Logsdail, A. J.; Woodley, S. M.; Senn, H. M.; Sherwood, P.; Catlow, C. R. A.; Sokol, A. A.; Keal, T. W. Multiscale QM/MM modelling of catalytic systems with ChemShell *Phys. Chem. Chem. Phys.* **2023**, *25*, 21816–21835.

(24) Frisch, M. J.; Trucks, G. W.; Schlegel, H. B.; Scuseria, G. E.; Robb, M. A.; Cheeseman, J. R.; Scalmani, G.; Barone, V.; Petersson, G. A.; Nakatsuji, H.; Li, X.; Caricato, M.; Marenich, A. V.; Bloino, J.; Janesko, B. G.; Gomperts, R.; Mennucci,

B.; Hratchian, H. P.; Ortiz, J. V.; Izmaylov, A. F.; Sonnenberg, J. L.; Williams; Ding, F.; Lipparini, F.; Egidi, F.; Goings, J.; Peng, B.; Petrone, A.; Henderson, T.; Ranasinghe, D.; Zakrzewski, V. G.; Gao, J.; Rega, N.; Zheng, G.; Liang, W.; Hada, M.; Ehara, M.; Toyota, K.; Fukuda, R.; Hasegawa, J.; Ishida, M.; Nakajima, T.; Honda, Y.; Kitao, O.; Nakai, H.; Vreven, T.; Throssell, K.; Montgomery Jr., J. A.; Peralta, J. E.; Ogliaro, F.; Bearpark, M. J.; Heyd, J. J.; Brothers, E. N.; Kudin, K. N.; Staroverov, V. N.; Keith, T. A.; Kobayashi, R.; Normand, J.; Raghavachari, K.; Rendell, A. P.; Burant, J. C.; Iyengar, S. S.; Tomasi, J.; Cossi, M.; Millam, J. M.; Klene, M.; Adamo, C.; Cammi, R.; Ochterski, J. W.; Martin, R. L.; Morokuma, K.; Farkas, O.; Foresman, J. B.; Fox, D. J. Wallingford, CT, 2009.

(25) Elstner, M.; Porezag, D.; Jungnickel, G.; Elsner, J.; Haugk, M.; Frauenheim, T.; Suhai, S.; Seifert, G. Self-consistent-charge density-functional tight-binding method for simulations of complex materials properties *Phys. Rev. B* **1998**, *58*, 7260–7268.

(26) Guest, M. F., Elena, A. M., & Chalk, A. B. G. (2019). DL_POLY - A performance over view analysing, understanding and exploiting available HPC technology. *Mol. Sim.* **47**, 194–227.

(27) Liu, D. C.; Nocedal, J. On the limited memory BFGS method for large scale optimization *Math.Progr.* **1989**, *45*, 503–528.

(28) Liao, R.-Z.; Thiel, W. Convergence in the QM-only and QM/MM modeling of enzymatic reactions: A case study for acetylene hydratase *J. Comput. Chem.* **2013**, *34*, 2389–2397.

(29) Gomez, H.; Polyak, I.; Thiel, W.; Lluch, J. M.; Masgrau, L. Retaining Glycosyltransferase Mechanism Studied by QM/MM Methods: Lipopolysaccharyl-α-1,4-galactosyltransferase C Transfers α-Galactose via an Oxocarbenium Ion-like Transition State *J. Am. Chem. Soc.* **2012**, *134*, 4743–4752.

(30) G. Santra, R. Calinsky, J. M. L. Martin, Benefits of Range-Separated Hybrid and Double-Hybrid Functionals for a Large and Diverse Data Set of Reaction Energies and Barrier Heights, *J. Phys. Chem. A* **2022**, *126*, 5492–5505

(31) Fu, Y.; Bernasconi, L.; Liu, P. Ab Initio Molecular Dynamics Simulations of the SN1/SN2 Mechanistic Continuum in Glycosylation Reactions *J. Am. Chem. Soc.* **2021**,

(32) Kühne, T. D.; Iannuzzi, M.; Del Ben, M.; Rybkin, V. V.; Seewald, P.; Stein, F.; Laino, T.; Khaliullin, R. Z.; Schütt, O.; Schiffmann, F.; Golze, D.; Wilhelm, J.; Chulkov, S.; Bani-Hashemian, M. H.; Weber, V.; Borštnik, U.; Taillefumier, M.; Jakobovits, A. S.; Lazzaro, A.; Pabst, H.; Müller, T.; Schade, R.; Guidon, M.; Andermatt, S.; Holmberg, N.; Schenter, G. K.; Hehn, A.; Bussy, A.; Belleflamme, F.; Tabacchi, G.; Glöß, A.; Lass, M.; Bethune, I.; Mundy, C. J.; Plessl, C.; Watkins, M.; VandeVondele, J.; Krack, M.; Hutter, J. CP2K: An electronic structure and molecular dynamics software package - Quickstep: Efficient and accurate electronic structure calculations *J. Chem. Phys.* **2020**, *152*, 194103.

(33) Tribello, G. A.; Bonomi, M.; Branduardi, D.; Camilloni, C.; Bussi, G. PLUMED 2: New feathers for an old bird *Comput. Phys. Commun.* **2014**, *185*, 604–613.

(34) Perdew, J. P.; Burke, K.; Ernzerhof, M. Generalized Gradient Approximation Made Simple *Phys. Rev. Lett.* **1996**, *77*, 3865–3868.

(35) Piniello, B.; Lira-Navarrete, E.; Takeuchi, H.; Takeuchi, M.; Haltiwanger, R. S.; Hurtado-Guerrero, R.; Rovira, C. Asparagine Tautomerization in Glycosyltransferase Catalysis. The Molecular Mechanism of Protein O-Fucosyltransferase 1 *ACS Catal.* **2021**, *11*, 9926–9932.

(36) Calvelo, M.; Males, A.; Alteen, M. G.; Willems, L. I.; Vocadlo, D. J.; Davies, G. J.; Rovira, C. Human O-GlcNAcase Uses a Preactivated Boat-skew Substrate Conformation for Catalysis. Evidence from X-ray Crystallography and QM/MM Metadynamics *ACS Catal.* **2023**, *13*, 13672–13678.

(37) Laun, J.; Vilela Oliveira, D.; Bredow, T. Consistent gaussian basis sets of double- and triple-zeta valence with polarization quality of the fifth period for solid-state calculations. *J. Comput. Chem.* **2018**, *39*, 1285–1290.

(38) Goedecker, S.; Teter, M.; Hutter, J. Separable dual-space Gaussian pseudopotentials. *Phys. Rev. B* **1996**, *54*, 1703–1710.

(39) Cremer, D.; Pople, J. A. General definition of ring puckering coordinates. *J. Am. Chem. Soc.* **1975**, *97*, 1354–1358.

(40) Ensing, B.; Laio, A.; Parrinello, M.; Klein, M. L. A Recipe for the Computation of the Free Energy Barrier and the Lowest Free Energy Path of Concerted Reactions *J. Phys. Chem. B* **2005**, *109*, 6676–6687.

(41) Marcos-Alcalde, I.; López-Viñas, E.; Gómez-Puertas, P. MEPSAnd: minimum energy path surface analysis over n-dimensional surfaces *Bioinformatics* **2020**, *36*, 956–958.

(42) Bolhuis P. G., Chandler D., Dellago C., Geissler P. L. Transition path sampling: throwing ropes over rough mountain passes, in the dark. *Annu Rev Phys Chem*. **2002**, *53*, 291-318.

(43) Johnson, E. R.; Keinan, S.; Mori-Sanchez, P.; Contreras-Garcia, J.; Cohen, A. J.; Yang, W. Revealing Noncovalent Interactions *J. Am. Chem. Soc.* **2010**, *132*, 6498–6506.

(44) Boto, R. A.; Peccati, F.; Laplaza, R.; Quan, C.; Carbone, A.; Piquemal, J.-P.; Maday, Y.; Contreras-García, J. NCIPLOT4: Fast, Robust, and Quantitative Analysis of Noncovalent Interactions *J. Chem. Theory Comput.* **2020**, *16*, 4150–4158.

(45) Humphrey, W.; Dalke, A.; Schulten, K. VMD - Visual Molecular Dynamics *J. Mol. Graph.* **1996**, *14*, 33–38.

(46) (a) Grin, Y.; Savin, A.; Silvi, B. In *The Chemical Bond: Fundamental Aspects of Chemical Bonding*; Frenking, G., Shaik, S., Eds.; Wiley-VCH: Weinheim, 2014, p 345–382. (b) Savin, A.; Nesper, R.; Wengert, S.; Fässler, T. F. ELF: The Electron Localization Function *Angew. Chem. Int. Ed.* **1997**, *36*, 1808–1832.

(47) (a) Gara, G.; Hurtado, J.; Pedrón, M.; García, L.; Reyes, E.; Sánchez-Díez, E.; Tejero, T.; Carrillo, L.; Merino, P.; Vicario, J. L. Organocatalytic Enantioselective Vinylcyclopropane-Cyclopentene (VCP-CP) Rearrangement *Angew. Chem. Int. Ed.* **2023**, *62*, e202302416. (b) Ortega, A.; Manzano, R.; Uria, U.; Carrillo, L.; Reyes, E.; Tejero, T.; Merino, P.; Vicario, J. L. Catalytic Enantioselective Cloke–Wilson Rearrangement *Angew. Chem. Int. Ed.* **2018**, *57*, 8225–8229. (c) Capel, E.; Rodriguez-Rodriguez, M.; Uria, U.; Pedron, M.; Tejero, T.; Vicario, J. L.; Merino, P. Absence of Intermediates in the BINOL-derived Mg(II)/Phosphate-Catalyzed Desymmetrizative Ring Expansion of 1-Vinylcyclobutanols *J. Org. Chem.* **2022**, *87*, 693–707.

(48) Fang, D.; Chaudret, R.; Piquemal, J. P.; Cisneros, G. A. Toward a Deeper Understanding of Enzyme Reactions Using the Coupled ELF/NCI Analysis: Application to DNA Repair Enzymes *J Chem. Theory Comput.* **2013**, *9*, 2156–2160.

(49) (a) Lu, T.; Chen, F. Multiwfn: A multifunctional wavefunction analyzer *J. Comput. Chem.* **2012**, *33*, 580–592. (b) Lu, T. A comprehensive electron wavefunction analysis toolbox for chemists, Multiwfn *J. Chem. Phys.* **2024**, *161*, 082503.
